# Genetic Differentiation of Geographically Overlapping Sister Species of *Eucalyptus* in Northern Australia

**DOI:** 10.1002/ece3.71454

**Published:** 2025-06-23

**Authors:** Harvey K. Orel, Todd G. B. McLay, Daniel J. Murphy, David J. Cantrill, Frank Udovicic, Patrick S. Fahey, Donald C. Franklin, Donna Lewis, Philip G. Docherty, Adam White, Michael J. Bayly, Rachael M. Fowler

**Affiliations:** ^1^ School of BioSciences, the University of Melbourne Parkville Victoria Australia; ^2^ National Biodiversity DNA Library Environomics, NCMI, CSIRO Parkville Victoria Australia; ^3^ Royal Botanic Gardens Victoria Melbourne Victoria Australia; ^4^ Department of the Environment, Tourism, Science and Innovation Queensland Herbarium and Biodiversity Science Toowong Queensland Australia; ^5^ Queensland Alliance for Agriculture and Food Innovation, the University of Queensland St Lucia Queensland Australia; ^6^ Research Institute for Environment & Livelihoods, Charles Darwin University Darwin Northwest Territories Australia; ^7^ Terrestrial Ecosystem Research Network (TERN) University of Adelaide Adelaide South Australia Australia; ^8^ Independent Scholar Cable Beach Western Australia Australia; ^9^ Australian National Herbarium, Centre for Australian National Biodiversity Research Canberra Australian Capital Territory Australia

**Keywords:** Australia, *Eucalyptus*, *Eudesmia*, introgression, phylogeography, sister speciation

## Abstract

In the large genus *Eucalyptus*, which dominates most of Australia's open forests and woodlands, genetic studies commonly show signs of introgression between closely related, co‐occurring species. Here we assessed genetic variation in two sister species with geographically overlapping distributions in northern Australia. One species, *Eucalyptus tetrodonta*, is a dominant species in many lowland savannas. It is found on gravelly red‐lateritic to sandy soils, with a large distribution spanning over 2000 km east‐to‐west and 1000 km north‐to‐south, from the Kimberley region of Western Australia to northern Queensland. The other, *E. megasapala*, was taxonomically separated from *E. tetrodonta* in 2006 on the basis of its larger sepals, prominently ribbed buds and operculum, and fruit and peduncle shape. It typically occurs on rocky substrates in northeast Queensland, within the range of *E. tetrodonta*, where the two species can form stands within a few hundred metres of one another. Contrary to expectations, DArTseq genotyping showed strong differentiation between the two species (*F*_ST_: 0.28) and little evidence of genetic admixture. Analyses of data from the Australasian Virtual Herbarium showed significant differences in flowering times between the two species. SNP outlier analyses identified multiple loci potentially under selection that are associated with differences between the two species, including the gene FT‐interacting protein 7 (FTIP7), which is known to modulate flowering time in plants. Based on current data, it is unclear whether differentiation of these species is the product of parapatric/sympatric speciation or if it was allopatric with secondary geographic overlap. Within *E. tetrodonta*, genetic variation showed a strong signal of isolation‐by‐distance, with an east–west trend in the pattern of genetic relatedness. The most substantial genetic break in *E. tetrodonta* was associated with the Carpentarian Gap, a region of seasonally arid, alluvial plains on the southern margin of the Gulf of Carpentaria known as a biogeographic barrier for other Australian biota.

## Graphical abstract

We assessed genetic variation in two closely related *Eucalyptus* species, *E. tetrodonta* and *E. megasapala*, finding strong genetic differentiation and little introgression despite geographic overlap. SNP outlier analyses identified loci under selection, and a genetic break in *E. tetrodonta* linked to the Carpentarian Gap, suggesting complex speciation processes.

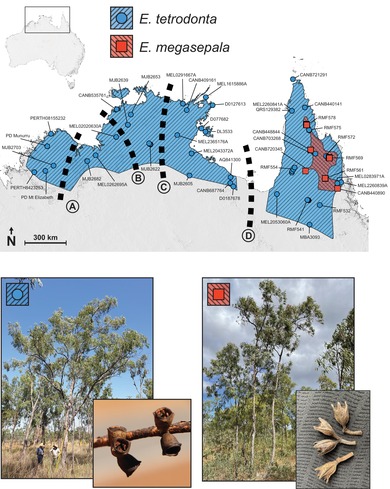

## 1 Introduction

An evolutionary pattern is emerging in some diverse, temperate tree genera, including *Quercus* (oaks), *Salix* (willows), *Pinus* (pines), and *Eucalyptus* (gum trees) (Keim et al. 1989; Hardig et al. 2000; Thorsson et al. 2007; Willyard et al. 2009; Hipp et al. 2019; Li et al. 2021; Zhang et al. 2024). These genera are characterised by a high level of species diversity and a wide range of environmental and genetic variation within species (Cannon and Petit 2020; Crowl et al. 2020; López de Heredia et al. 2020). They can have high levels of hybridisation between related species, suggesting leaky species boundaries and incomplete reproductive isolation (Cannon and Petit 2020; Buck and Flores‐Rentería 2022; Ma et al. 2024). Groups of species with high levels of within‐species variation and high levels of hybridisation between species have been referred to as ‘syngameons’ (Cannon and Petit 2020; Sanderson et al. 2023). Such a strategy may be an advantage in long‐lived trees, as high levels of intraspecific diversity and low or weak reproductive barriers between species may provide adaptive advantages to changing climatic and environmental conditions, as the genetic entities can produce offspring that are extremely plastic (Wang et al. 2020; Cannon and Petit 2020). However, the question remains of how such a high level of species diversity can be generated and maintained when species are not reproductively isolated.

In Australia, there are over 700 species of *Eucalyptus*, which dominate many vegetation types (APC 2024; Nicolle 2024). *Eucalyptus* species are known to hybridise extensively (Pryor 1953; Griffin et al. 1988; McKinnon et al. 2004), especially those within the same subgenera, sections, or series (Larcombe et al. 2015). Inferring relationships between eucalypt species can be complex due to a combination of recent/contemporary hybridisation and plastid capture (e.g., McKinnon et al. 2004, 2010; Pollock et al. 2013, 2015; Nevill et al. 2014), and earlier phylogenetic studies were limited by the molecular tools available at the time (Bayly 2016). High‐throughput sequencing technologies have allowed greater insight into the extent of hybridisation, including patterns of both plastid (Schuster et al. 2018; Fahey et al. 2021) and nuclear DNA introgression (Rutherford et al. 2018, 2019, 2021, 2023; Collins et al. 2019; Murray et al. 2019; von Takach Dukai et al. 2019; Fahey et al. 2022a, 2022b), among species that co‐occur or almost so.

Speciation in syngameons and plants in general is considered to occur primarily via allopatry, where populations of a species become separated by edaphic, climatic, or physical barriers (Binks et al. 2021; Hernández‐Hernández et al. 2021). This separation prevents gene flow between populations, resulting in reproductive isolation and eventually speciation and associated fixed genetic and morphological differences between species. However, if speciation does not lead to strong reproductive barriers, secondary contact between populations of those species can provide opportunities for genetic exchange and hybridisation, resulting in co‐occurring species that show levels of genetic admixture (Buck et al. 2023; Cannon and Petit 2020).

Sympatric speciation (occurring without geographic barriers) is considered to be less common and more difficult to identify and demonstrate than allopatric speciation (Fitzpatrick et al. 2008; Papadopulos et al. 2011; Bird et al. 2012; Richards et al. 2019; Sun et al. 2022). Nonetheless, sympatric speciation has been postulated in several different organisms, predominantly in animals (Kautt et al. 2020; Sutra et al. 2019), but more evidence is emerging for sympatric speciation in plants (Osborne et al. 2019, 2020; Tavares et al. 2022). Under allopatry, divergence between populations can be promoted through factors such as genetic drift (which can be impacted by generation times and population sizes) and environmental selection, without the mitigating impact of gene flow to reconnect the lineages (Sobel et al. 2010). However, in sympatry, the mechanisms to enable speciation must promote divergence in the face of possible gene flow and therefore have to be significantly impactful over relatively shorter time periods (Wellborn and Langerhans 2015). A number of isolating mechanisms have been implicated as leading to sympatric speciation in plants, including both pre‐zygotic mechanisms (e.g., phenology (Osborne et al. 2019, 2020), pollinator preference (Chen 2013), pollen‐tube inhibition (Pellegrino 2016)) and post‐zygotic mechanisms (e.g., chromosomal rearrangements (Wang et al. 2020), chromosome duplications and polyploidy (Tate et al. 2005), reduced germination success (de Holanda et al. 2015)). These mechanisms may act in concert to enhance and maintain reproductive isolation. In eucalypts, allopatric speciation has generally been assumed or inferred (Ladiges 1997; McGowen et al. 2001), and although parapatric differentiation of ecotypes has been inferred (Foster et al. 2007), examples of sympatric speciation have not been substantiated.

**FIGURE 1:**
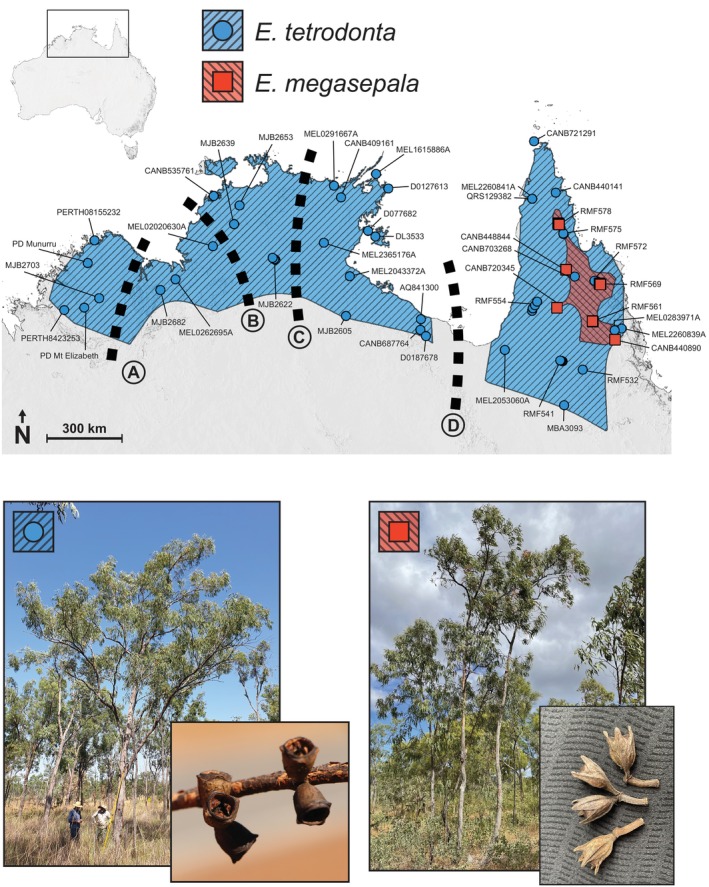
Geographic ranges of *Eucalyptus tetrodonta* and *E. megasepala* (diagonally‐lined regions), including collection sites (circles or squares) for samples included in this study. Thick dashed lines indicate notable biogeographic barriers identified by Edwards et al. (2017): (A) Ord Basin; (B) Daly River Plains; (C) Arnhem Land Plateau; (D) Carpentarian Gap. Photographs compare habit and fruit morphology of each species. Voucher specimen numbers or collector numbers are indicated for all collecting sites (details in Table S1). Photos: F. Udovicic and M. Bayly (*E. tetrodonta*); D. Nicolle (*E. megasepala*; CC BY‐NC 4.0, https://www.inaturalist.org/observations/96957531).

*Eucalyptus* subg. *Eudesmia* is a clade of 22 species that occurs across large parts of western, central, and northern Australia. A high level of introgression and hybridisation has been detected using phylogenomic analyses (McLay et al. 2023), suggesting that gene flow between species is common within this subgenus. The centre of phylogenetic diversity for *E*. subg. *Eudesmia* is in northern Queensland, although species diversity is highest in Western Australia (McLay et al. 2023). The most widespread species in the subgenus is *E. tetrodonta*, which occurs in Western Australia, the Northern Territory, and Queensland in the monsoonal tropics of northern Australia (Figure 1). It occurs on a range of soil types including red bauxitic laterites, red earths, infertile sand plains, and sand on sandstone, in pure stands or mixed with a wide variety of other species (Fox et al. 2001; Bean 2006; Franklin 2022). In 2006, some Queensland populations previously included in *E. tetrodonta* were described as a new species, *E. megasepala* (Bean 2006). Morphologically, the two species are similar, with *E. megasepala* being differentiated from *E. tetrodonta* by larger sepals, prominently ribbed buds, conical opercula, strongly flattened peduncles, and fruits that are square in cross‐section (Bean 2006). Additionally, in the region in which their ranges overlap, the two species display distinct fine‐scale ecological preferences, with *E. megasepala* often occurring on rocky rises, and *E. tetrodonta* tending to occur on lower slopes. Despite these differences, the range of *E. megasepala* is entirely nested within the range of *E. tetrodonta*, and plants of the two species can be found within metres of one another (RMF, personal observation) in discrete, adjacent stands (Franklin 2022). Our recent phylogenomic analysis (McLay et al. 2023) confirmed that *E. megasepala* and *E. tetrodonta* formed a distinct clade within *E*. subg. *Eudesmia* (taxonomically recognised at the level of series; Bayly et al. 2023), but resolved *E. megasepala* nested among Queensland samples of *E. tetrodonta*, questioning the distinctiveness of the more recently described species.

To investigate the relationship between the geographically overlapping sister species, *E. megasepala* and *E. tetrodonta*, and the genetic structure of *E. tetrodonta* across the entire species' range, we performed Diversity Arrays Technology sequencing (DArTseq) on 94 samples. DArTseq is a genome reduction method that allows for the detection of a high number of informative SNPs from across the genome (Melville et al. 2017). DArTseq has been widely applied across many plant systems for studies of genetic diversity, population structure, hybridization, speciation, phylogeography, etc. (Steane et al. 2015; Edet et al. 2018; Rutherford et al. 2018; Rutherford 2020; Fahey et al. 2022a; Rosser et al. 2023; Palsson et al. 2024; Vašut et al. 2024). Contrary to our expectations, tests of diversity and differentiation identified strong genetic distinction between the two species and low evidence of gene flow. This result was investigated more thoroughly using genetic outlier analysis to identify SNPs that strongly differentiated the two species (Ahrens et al. 2018; Kidner et al. 2021) and ecological data in the form of broad‐scale environmental variables and flowering time data collected from herbarium specimen metadata. We also detected very low genetic structure in *E. tetrodonta*, despite this species occurring in a wide geographic range across several potential biogeographic barriers (Edwards et al. 2017). Here we identify high genetic differentiation between co‐occurring *Eucalyptus* species that are strongly supported as sister taxa.

## 2 Methods

### 2.1 Study Species and Sampling

*Eucalyptus tetrodonta* and *E. megasepala* are two sister species (McLay et al. 2023) with geographic distributions in northern Australia. *Eucalyptus tetrodonta* is common and widespread, spanning over 1000 km north‐to‐south and over 2000 km east‐to‐west from the Kimberley region of Western Australia to the Cape York Peninsula in Queensland (Figure 1). It occurs across a number of biogeographic barriers in northern Australia, including the Carpentarian Gap, around which there is a disjunction in its distribution (see Figure 1). *Eucalyptus megasepala* is restricted in distribution to far northeastern Queensland, where it overlaps in distribution with *E. tetrodonta* on Cape York Peninsula. Both species are upright, fibrous‐barked trees with lanceolate to falcate, opposite to sub‐opposite adult leaves, and the two species are hard to distinguish if buds, flowers, or fruits (capsules) are lacking (Slee et al. 2020). However, there are several key differences between the two species. *Eucalyptus tetrodonta* has capsules that are round in cross‐section with four small teeth (remnant sepals), flower buds with a dome‐shaped cap and at most faint ribbing, and sepals that are 1–3 mm long; it flowers from July to October. In contrast, *E. megasepala* has capsules that are square in cross‐section with very prominent ribs, flower buds with a cap that tapers to a blunt tip and very prominent ribbing, and sepals that are 5–9 mm long; it flowers from April to July. In addition, *E. tetrodonta* and *E. megasepala* tend to occupy different habitats in their geographic area of overlap; *E. tetrodonta* is more common in more fertile downslopes environments, while *E. megasepala* is more common in less fertile soils on upslopes environments. However, the two species occur within metres of one another in some parts of their range, especially on the extensive Laura sandstone (Franklin 2022).

For this study, leaf material was collected from 94 plant specimens, including 73 *E. tetrodonta* samples, 18 *E. megasepala* samples, and one each of three outgroup species (*E. chartaboma*, *E. eudesmioides* , *E. similis* —see Table S1), representing distinct clades of *E*. subg. *Eudesmia* (McLay et al. 2023). Sampling included both field and herbarium collections. Where possible, population‐level sampling was completed across the range of each species (10 populations of *E. tetrodonta* and three populations of *E. megasepala*). Most population samples consisted of five individuals, collected approximately 1 km apart to reduce the likelihood of sibling relatedness between plants, or of stems being genetically identical root suckers; *E. tetrodonta* being one of only two eucalypt species known to produce root suckers (Lacey and Whelan 1976; Franklin et al. 2010). Leaf tissue was provided to Diversity Arrays Technology Pty Ltd. (Canberra, Australia) for DNA extraction and sequencing using the DArTseq platform. Genotype data were generated from total genomic DNA using proprietary analytical pipelines (DArT Pty Ltd).

### 2.2 Data Filtering

Following sequencing, two sets of genotype data were requested from DArT; one dataset including all samples (‘total data’), and one dataset with outgroup samples excluded (‘no outgroup data’). This format was specified to maximise the number of ingroup‐specific SNPs retained during DArT pipeline filtering for analyses that did not require outgroups. Of the 94 and 91 samples initially included in each of these respective datasets (total data/no outgroup data), five herbarium samples failed sequencing (*E. tetrodonta*—MBA3093.2, MEL0283971A, MEL1615886A, CANB 721291; *E. megasepala*—CANB 440890) and were excluded from the rest of the study.

**FIGURE 2:**
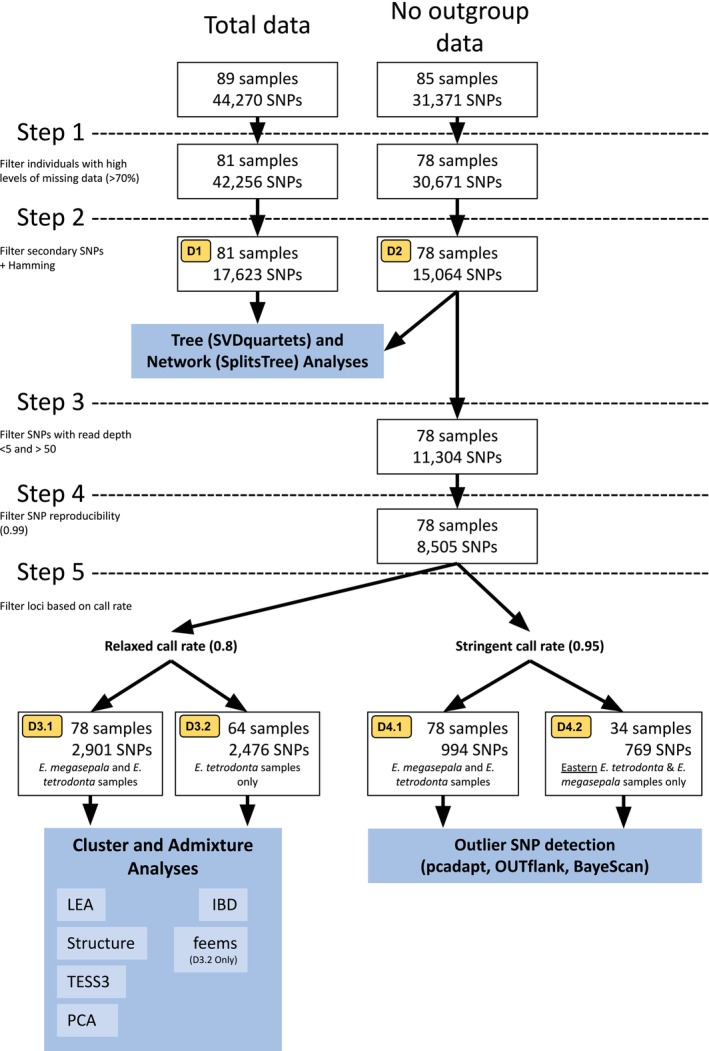
Flowchart of data processing steps with resultant datasets (yellow boxes, e.g., D3.2 = Dataset 3.2) and methods of analysis (blue boxes).

The datasets received from DArT consisted of 44,270 SNPs (total data) and 31,371 SNPs (no outgroup data). Both datasets were subject to SNP filtering in the R package *dartR* v.2.9.7 (Mijangos et al. 2022) (see Figure 2 for a flowchart detailing the filtering process). Both datasets were initially screened for samples with high levels (> 70%) of missing data and eight ingroup samples (CANB720345, D0077682, D0187678, MEL2020630A, MEL2043372A, MEL2260840A, MEL2260841A, RMF569.5) were removed from both datasets resulting in a ‘total data’ set of 81 samples (Figure 2; Dataset 1) and a ‘no outgroup data’ set of 78 samples (Figure 2; Dataset 2). Both datasets were then filtered to remove instances of more than one SNP identified from a single locus (i.e., potentially linked SNPs) using the function ‘gl.filter.secondaries’, and potentially duplicated or paralogous loci using ‘gl.filter.hamming’ (both with default parameters).

For further analyses of relationships between species and populations, the ‘no outgroup data’ (Figure 2; Dataset 2) set was subjected to further filtering steps, retaining SNPs with a read depth between 5 and 50, and reproducibility (RepAvg) value ≥ 0.99. We then filtered the data using two different call rate (CR: the proportion of samples with no missing data for a SNP) thresholds for conducting separate population genetic analyses capable of handling different levels of missing data. For this, we used thresholds of CR ≥ 0.80 and CR ≥ 0.95.

The data produced from the CR ≥ 0.80 filtering were used to form two datasets, Dataset 3.1 and Dataset 3.2 (Figure 2), for input into PCA, admixture, and isolation‐by‐distance analyses. Dataset 3.1 included all samples of *E. megasepala* and *E. tetrodonta*; we also removed all *E. megasepala* samples from Dataset 3.1 (also recalculating locus statistics and removing monomorphic loci) to produce Dataset 3.2, which included only samples of *E. tetrodonta*.

The data produced from the CR ≥ 0.95 filtering was used to form two datasets, Dataset 4.1 and Dataset 4.2 (Figure 2), for input to tests aimed at detecting SNPs under divergent selection between *E. megasepala* and *E. tetrodonta*. Dataset 4.1 included all samples of *E. megasepala* and *E. tetrodonta*, and Dataset 4.2 included only samples of *E. tetrodonta* collected from a geographic area that could be considered overlapping with that of *E. megasepala* (those at most 222 km from any *E. megasepala* collections recorded on AVH 2023, and cleaned per the *Analysis of flowering time* section).

### 2.3 Phylogenetic Analyses

We undertook phylogenetic analyses of the ‘total data’ set (Figure 2; Dataset 1) and the ‘no outgroup data’ set (Figure 2; Dataset 2) using tree and network approaches. For both datasets, we ran SVDquartets (Chifman and Kubatko 2014) in PAUP* v.4.0a (Swofford 2002) to produce phylogenetic trees using a site‐based coalescent approach. For this, SNP matrices were formatted to a single sequence per sample, with ambiguity codes used for heterozygous sites, and samples were treated as individuals (i.e., not grouped by species or population). We exhaustively sampled all quartets, using Quartet FM (Reaz et al. 2014) for quartet assembly, and inferred node support with 100 nonparametric bootstrap replicates. To generate phylogenetic networks, we imported the SNP matrices into SplitsTree4 (Huson and Bryant 2006) and viewed the network under default import settings.

### 2.4 Ordinations, Diversity Measures, Admixture and Isolation‐By‐Distance

To investigate patterns of genetic variation and structure in *E. megasepala* and *E. tetrodonta*, we ran several analyses using Dataset 3.1 and Dataset 3.2 (Figure 2). For each dataset, we carried out successive k‐means clustering using the ‘find. clusters’ function of *adegenet* v.2.1.10 (Jombart 2008), testing values of *k* from 1 to 40, retaining 100 principal components and evaluating goodness‐of‐fit for each *k*‐value using the Bayesian Information Criterion (BIC). We also ran Pearson principal component analyses (PCAs) on each dataset using the ‘gl.pcoa’ function in *dartR* with default settings. To further examine genetic diversity and differentiation between species and populations of *E. megasepala* and *E. tetrodonta*, we calculated unbiased expected heterozygosity (u*H*_e_), a metric that accounts for variation in sample size between populations, from Dataset 3.1 using the ‘gl.report.heterozygosity’ function in *dartR*. We also calculated *F*_ST_ estimates between the species and populations (excluding singleton specimens from herbarium collections) from Dataset 3.1 using the ‘gl.fst.pop’ function and 100 bootstrap replicates in *dartR*.

Genetic structure in Dataset 3.1 and Dataset 3.2 was first examined using the R package LEA v.3.8.0 (Frichot and François 2015) to estimate admixture coefficients for each sample using sparse nonnegative matrix factorisation (snmf) with the function ‘snmf’. We ran snmf for values of *k* from 1 to 10, with 40 replicate runs per *k*‐value, and estimated cross‐entropy. The optimal value of *k* for each dataset was identified by the interpretation of cross‐entropy scores, with the value of *k* with the lowest cross‐entropy score considered optimal. To supplement our assessment of admixture with LEA and test for consistent results across different methods, we performed additional analyses aimed at detecting population structure and admixture using the program Structure v.2.3.4 (Pritchard et al. 2000). In Structure, we conducted 20 replicate runs for each value of *k* from 1 to 7 using 300,000 MCMC cycles with a burn‐in of 100,000 cycles. We used Structure Harvester v.0.6.94 (Earl and vonHoldt 2012) to investigate optimal values of *k*, following the method of Evanno et al. (2005), and runs were combined in CLUMPAK (Kopelman et al. 2015) to obtain ancestry proportions for major clusters of each *k*‐value. Ancestry proportions for *k* = 2–7 were then visualised as bar plots. The non‐spatially explicit models employed by LEA snmf and Structure are known to be susceptible to producing inaccurate results under IBD, usually in the form of detecting additional artificial genetic clusters (Frantz et al. 2009; Meirmans 2012; Perez et al. 2018). To determine whether IBD was present in our Datasets 3.1 and 3.2, we conducted Mantel tests and generated IBD plots for each dataset using the ‘gl.ibd’ function of *dartR* with 9999 permutations to calculate pairwise genetic and geographic distances between individual samples using Euclidean distance.

Following the detection of IBD in *E. tetrodonta* (Dataset 3.2), we ran *FEEMS* (Marcus et al. 2021) to estimate effective migration surfaces and visualise spatially heterogeneous IBD across the range of *E. tetrodonta*. For this, we converted Dataset 3.2 to the PLINK format with PLINK v.1.90b7 (Purcell et al. 2007; Chang et al. 2015) and the ‘gl2plink’ function in *dartR*. We used the R package *dggridR* v.3.0.0 (Barnes et al. 2017) to generate two triangular grids covering the species' distribution with differing cell sizes of 389 km^2^ and 1557 km^2^. We conducted *FEEMS* runs using both grids, performing leave‐one‐out cross‐validation over a grid of tuning parameter values (λ; which controls the strength of the penalisation placed on the output of the migration surface) to select the optimal λ value for each run.

Finally, we used the R package *triangulaR* v.0.0.0.9000 (Wiens and Colella 2024) to calculate hybrid indices and interclass heterozygosity between populations of *E. megasepala* and *E. tetrodonta* from Dataset 4.1, testing five allele frequency difference thresholds (0.5, 0.6, 0.7, 0.8, 0.9). Samples from herbarium vouchers for which a single specimen was sampled at a locality were treated as separate, singleton populations.

### 2.5 Detection of Outlier Loci

To identify loci potentially under selection that may be associated with biological differences between *E. megasepala* and *E. tetrodonta*, we used three different programs for detecting outlier SNPs in Dataset 4.1 and Dataset 4.2. *Pcadapt* v.4.3.3 identifies *F*_ST_ outliers by ascertaining population structure using PCA and then determining associations between principal components and locus variation for each SNP, with the underlying assumption that loci experiencing divergent selection exhibit non‐typical relationships to principal components (Luu et al. 2017; Privé et al. 2020). We ran *pcadapt* using Mahalanobis distance to compute *p*‐values for SNPs with minor allele frequencies > 5% for 20 principal components (PCs). The optimal number of PCs was identified from an interpretation of the scree plot using Cattell's Rule (Cattell 1966). The R package *qvalue* v.2.30.0 (Storey et al. 2019) was then used to transform *p*‐values to *q*‐values, and SNPs with a false discovery rate (FDR) *q* < 0.05 were identified as outliers.

*OutFLANK* v.0.2 (Whitlock and Lotterhos 2015) calculates an *F*_ST_ measure of genetic differentiation for each locus and estimates a null χ^2^ distribution based on the neutral loci, then uses this as the null distribution to identify loci under selection (i.e., in the right tail) of the full χ^2^ distribution. We ran *OutFLANK* through *dartR* using the ‘gl.outflank’ function with default settings.

*BayeScan* v.2.1 (Foll and Gaggiotti 2008) estimates *F*_ST_ coefficients for two components, a population‐specific component (β) and a locus‐specific component (α), and considers loci to have departed from neutrality when the locus‐specific component is necessary to explain the observed pattern of diversity (i.e., when α is significantly different from zero). Positive values of α suggest divergent selection, while negative values suggest balancing selection. A reversible‐jump MCMC algorithm is then used to estimate the posterior probability of a neutral selection model (that does not include the α component) and a selection model (that includes the α component) for each locus. To run BayeScan, genlight objects for each dataset were converted to BayeScan format in *dartR* using the ‘gl2bayescan’ function, with samples assigned to populations based on their species. We then ran BayeScan twice using different values for prior odds; this parameter sets the prior odds for the neutral model, where a value of 1 is equivalent to assuming that for each locus the neutral model is equally as likely as the model including selection, and increasing this value increases the likelihood of the neutral model. Higher prior odds values tend to eliminate false positives at the expense of reducing the power to detect markers under selection, and the prior odds value has a decreasing influence with increasing dataset size. For the first run, we used the default prior odds value of 10 (recommended for hundreds of markers) with 10,000 iterations after a burn‐in of 200,000 iterations. For the second run, we used a more relaxed prior odds value of 2, with 10,000 iterations after a burn‐in of 200,000 iterations. For both runs, all other settings were left at their defaults, and any SNPs with *q‐*values < 0.2 (corresponding to allowing 10% of false positives) were treated as outliers.

### 2.6 Annotation of Outlier Loci

To investigate the identities of outlier loci and their potential relation to gene function, outlier SNP information (including SNP position, minor allele frequency, and other metadata) was exported to a vcf file from *dartR*. The outlier vcf files were imported into Geneious Prime 2023.2.1 (http://www.geneious.com/) and mapped to the annotated *E. grandis* genome (Myburg et al. 2014); loci that did not map to this reference genome during the initial DArT mapping procedure were excluded from further analyses. In Geneious, we extracted 750 bp of flanking sequence from both sides of the SNP to create contigs 1501 bp long. From the annotations in each contig, we recorded the SNP position (including whether the SNP was inside or outside a coding sequence (CDS) with the number of bases from the nearest CDS, and if inside a CDS, whether or not the SNP occurred inside an exon or intron), and *E. grandis* Gene ID, Protein ID, and Product. To associate gene ontology (GO) annotation terms to outlier SNPs using the Gene Ontology database (Carbon et al. 2009; Gene Ontology Consortium 2004), *E. grandis* Gene IDs were searched in Batch Entrez (https://www.ncbi.nlm.nih.gov/sites/batchentrez) to obtain gene names searchable on the UniProt ID mapping portal (https://www.uniprot.org/id‐mapping), whereafter we used the results returned by this portal to download GO IDs. REViGO (http://revigo.irb.hr) was then used to summarise and visualise the effects of GO terms across all outlier SNPs.

### 2.7 Environmental Variables

To compare the overlap in environmental conditions between the two species, we used all 19 bioclimatic variables from WorldClim 2 (Fick and Hijmans 2017) across the entire range of *E. tetrodonta* and eight soil attributes (AWC, available water capacity; BDW, bulk density; CLY, clay; ECE, effective cation exchange capacity; NTO, total nitrogen; pHc, pH CaCl_2_; PTO, total phosphorus; and SND, sand) from the Soil and Landscape Grid of Australia (Rossel et al. 2015). The soil attributes were limited to the range of *E. megasepala* and were downloaded using package slga (https://github.com/obrl‐soil/slga, accessed October 2023). Specimen data were downloaded for both species from the Australasian Virtual Herbarium (AVH; https://avh.chah.org.au/; accessed October 2023), and data were cleaned manually to remove records with erroneous data. Environmental variables were appended to the specimen data based on spatial coordinates using the packages *raster* (https://cran.r‐project.org/web/packages/raster/index.html, accessed October 2023) and *geodata* (https://cran.r‐project.org/web/packages/geodata/index.html, accessed October 2023). Pairwise regressions were used to identify autocorrelated relationships between variables, and the dataset was filtered so that variables were independent. The final set of bioclimatic and soil variables was compared across the range of *E. tetrodonta* with a PCA using the *factoextra* package (https://cran.r‐project.org/web/packages/factoextra/index.html, accessed October 2023).

### 2.8 Analysis of Flowering Time

Following the method of Osborne et al. (2020), we used herbarium data to estimate and compare flowering time in *E*. *megasepala* and *E. tetrodonta*. For this, all herbarium records for both species were downloaded from the AVH (accessed 2023). To subset these data to records where the individual tree represented by the voucher specimen was in flower, we retained only those records returning a match for the word “flower” under the Darwin Core (Wieczorek et al. 2012) categories ‘reproductiveCondition’ and ‘occurrenceRemarks’. Following this, records with missing geographic coordinate data (i.e., decimal latitude and longitude) and missing or incomplete collection date information (i.e., year, month, and day all required) were removed. To remove duplicate specimens held across different herbaria, we identified records with duplicate ‘locality’ information and duplicate ‘recordNumber’ and ‘recordedBy’ information, and retained only a single specimen from the duplicate set. The resulting data were then checked by eye, and any duplicates that had passed previous filtering steps were manually removed. The phenology and identity of specimens held at CANB (10/20 *E. megasepala* and 37/85 total *E. tetrodonta* records) were verified in‐person by TGBM; the identity of specimens at BRI (5/20 *E. megasepala* and 15/85 *E. tetrodonta*) had previously been verified by A.R. Bean. We classed each record as a single‐point observation of flowering time and converted the flowering date to days after 1 January of the collection year. To investigate patterns of flowering time with respect to geography, the *E. tetrodonta* dataset was divided into two additional datasets, based on locality, for comparison with *E. megasepala*. The first subset included only specimens collected from areas that could be considered to overlap with the range of *E. megasepala* (any *E. tetrodonta* specimens < 222 km from any *E. megasepala*); the second included only specimens collected from areas that could be considered to be non‐overlapping with the range of *E. megasepala* (any *E. tetrodonta* specimens > 222 km from any *E. megasepala*). To generate the ‘overlapping’ and ‘non‐overlapping’ subsets, the geographic range of *E. megasepala* was converted from point data to a polygon in QGIS v.3.16 using the Concave Hull v.2.0 plugin (https://plugins.qgis.org/plugins/concavehull/) and expanded outwards by ~222 km (2°) using the ‘Buffer’ tool. Records of *E. tetrodonta* falling within the buffered polygon were then extracted for the ‘overlapping’ subset using the ‘Clip’ tool, and the inverse of these was extracted for the ‘non‐overlapping’ subset. The distribution of dates from each *E. tetrodonta* dataset was compared with those of the *E. megasepala* dataset using Mann–Whitney U tests.

## 3 Results

### 3.1 Phylogenetic Inferences

**FIGURE 3:**
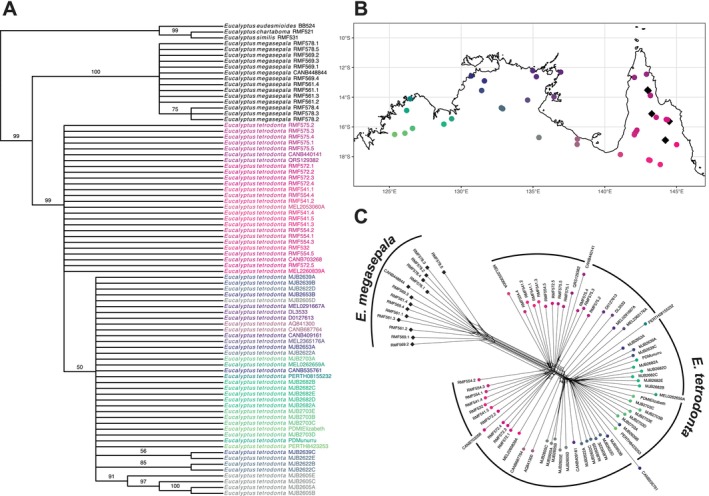
Results of phylogenetic analyses. (A) SVDquartets phylogeny generated from analysis of Dataset 1 (81 samples [3 outgroup], 17,623 unlinked SNPs), with branch support denoted by bootstrap values above branches and branches < 50% supported collapsed. (B) Map showing sample localities for *Eucalyptus tetrodonta* (circles) and *E. megasepala* (diamonds), with *E. tetrodonta* samples coloured by latitude and longitude and corresponding with tip colours in (A) and (C). (C) Neighbour‐Net network generated from Dataset 2 (78 samples [no outgroups], 15,064 unlinked SNPs).

The phylogenetic trees produced by SVDquartets analyses of Dataset 1 and Dataset 2 were topologically congruent and resolved *E. megasepala* and *E. tetrodonta* both on well‐supported branches (Bootstrap Support (BS): > 99% for the stem branches of each species across both datasets). The Dataset 1 tree (Figure 3A), which includes outgroup samples, resolved the two species as sister to each other with high support (BS: 99%). Overall, relationships between populations within each of these species were poorly resolved, although SVDquartets did recover a weakly supported clade (BS: 50%) of populations of *E. tetrodonta* from west of the Carpentarian Gap (Figure 3A,B).

The phylogenetic networks produced by SplitsTree showed *E. megasepala* and *E. tetrodonta* to be distinct from each other (Figure 3C), with each species forming well‐defined lineages with little reticulation between them. The network generated from Dataset 1 (not shown) showed evidence of separate reticulation between the outgroups and each of *E. megasepala* and Queensland populations of *E. tetrodonta*. Populations within each species were not clearly defined, although samples of *E. tetrodonta* tended to group based on geographic location (Figure 3C).

### 3.2 Patterns of Genetic Variation, Diversity and Structure

**FIGURE 4:**
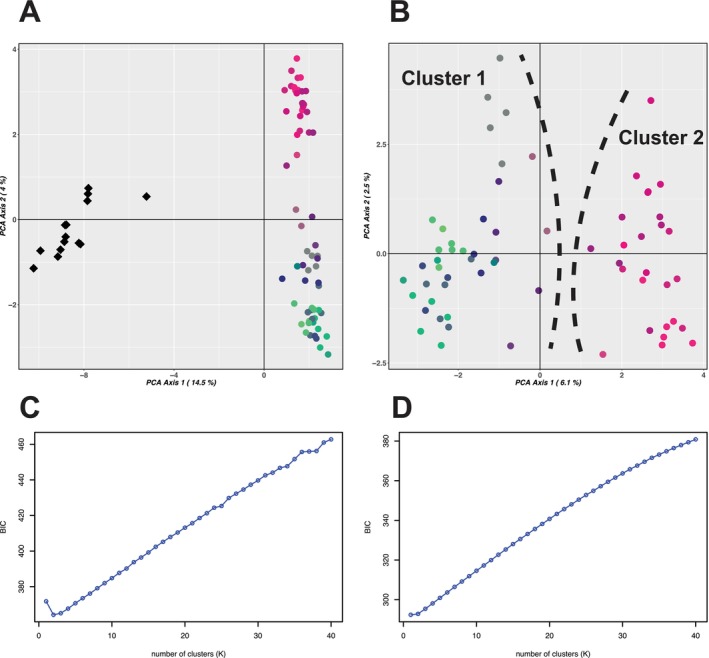
Results of *k*‐means clustering and PCA for Dataset 3.1 (*Eucalyptus megasepala* and *E. tetrodonta*; A, C) and Dataset 3.2 (*E. tetrodonta* only; B, D). (A) Plot of first two principal components, with two distinct genomic clusters detected by *k*‐means clustering (lowest BIC in C) corresponding to species (diamonds = *E. megasepala*, circles = *E. tetrodonta*); *E. tetrodonta* samples are coloured by latitude and longitude to correspond with Figure 3. (B) Plot of first two principal components for samples of *E. tetrodonta*, coloured by latitude and longitude to match Figure 3, with clusters for *k* = 2 separated by dashed lines, and plot showing support for *k* = 1 using *k*‐means clustering (D).

BIC scores for each *k*‐value tested by *k*‐means clustering varied across Dataset 3.1 and Dataset 3.2 (Fig. Figure 4C,D). For Dataset 3.1, the optimum value of *k* was identified as 2 (BIC: 364.2), with the clusters identified under this value corresponding to the species *E. megasepala* and *E. tetrodonta*. The PCA for Dataset 3.1 also retrieved two strongly distinct groups that corresponded with the two species (Figure 4A). The proportion of genetic variation explained by the first four and 10 principal components was 22.4% and 32.5%, respectively, with the first principal component accounting for 14.5% of the variation in the dataset and strongly separating *E*. *megasepala* and *E. tetrodonta* (Figure 4A). For Dataset 3.2, *k*‐means clustering returned the lowest BIC score for *k* = 1 (BIC: 292.4), with BIC scores steadily increasing for each value of *k* after this (Figure 4D). Under *k* = 2, the second‐best supported value by BIC (BIC: 292.9), the two resultant groups corresponded with *E. tetrodonta* samples from either side of the Carpentarian Gap (Figure S1). The PCA for Dataset 3.2 (Figure 4B) did weakly distinguish these groups, albeit with a lower proportion of variation explained by the first four (13.4%) and 10 (26.3%) principal components. In particular, the first and second principal components only accounted for 6.1% and 2.5% of variation, respectively.

We found similar levels of genetic diversity in *E. megasepala* (u*H*_e_: 0.14) and *E. tetrodonta* (u*H*_e_: 0.10). Likewise, genetic diversity between populations of *E. tetrodonta* was similar across its range (QLD: four populations, mean u*H*_e_: 0.094; NT: four populations, mean u*H*_e_: 0.082; WA: two populations, mean u*H*_e_: 0.087).

**FIGURE 5:**
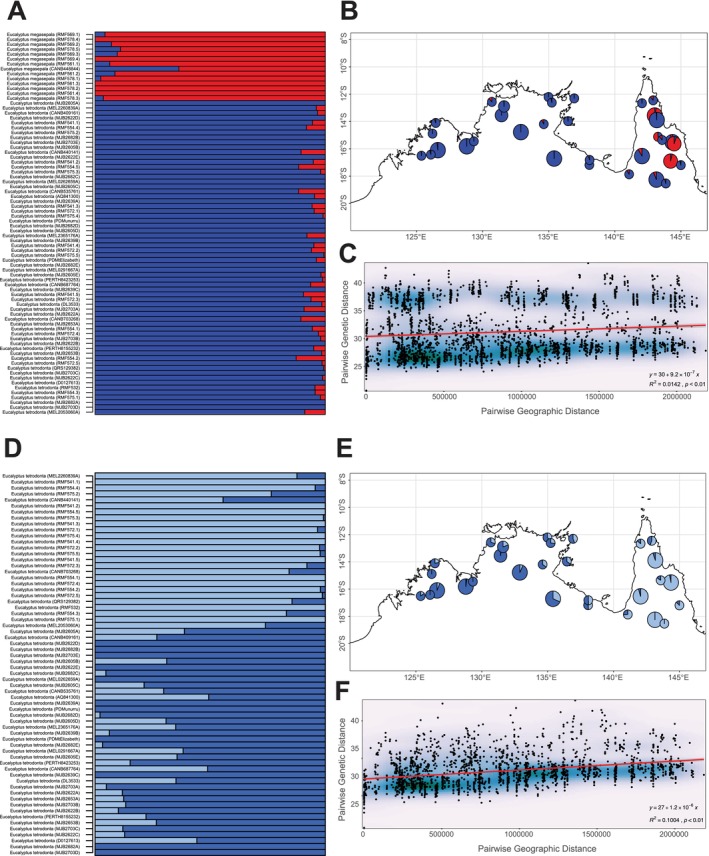
LEA admixture coefficients and isolation‐by‐distance plots for Dataset 3.1 (*Eucalyptus megasepala* and *E. tetrodonta*; A–C) and Dataset 3.2 (*E. tetrodonta* only; D–F). (A, D) Structure‐like barplots of ancestry coefficients for all individuals. (B, E) Mean population ancestry coefficients mapped as pie‐charts according to collection locality, with pies scaled according to individuals in each population (smallest *n* = 1, largest *n* = 5). (C, F) Isolation‐by‐distance plots, showing individual pairwise genetic distance against geographic distance.

Both methods used for identifying the optimal *k*‐value from results of LEA snmf and structure analyses suggested *k* = 2 as optimal for Dataset 3.1 and Dataset 3.2. For Dataset 3.1, the groups delineated under *k* = 2 for both analyses corresponded with *E. megasepala* and *E. tetrodonta* (Figure 5A; Figure S2); and genetic differentiation between the two species and clusters measured using *F*_ST_ was 0.28. Estimated ancestry coefficients showed low levels of admixture between the species, particularly for the LEA results, although one individual of *E. megasepala* (herbarium specimen CANB448844) showed a higher proportion of mixed ancestry (36% *E. tetrodonta*, 64% *E. megasepala*) than all other samples (Figure 5A). This sample was also placed closer to *E. tetrodonta* in the PCA for Dataset 3.1 (Figure 4A) but did not show greater levels of mixed ancestry in the structure analysis (Figure S2); it is likely that the PCA and LEA results for the sample were affected by the high percentage of missing data for this sample (38% missing) compared with other samples of *E. megasepala*. For Dataset 3.2, the groups delineated under *k* = 2 in LEA and structure analyses weakly corresponded to samples collected from either side of the Carpentarian Gap; however, we found large amounts of admixture between these groups, most notably among samples collected from the middle of the longitudinal range (Figure 5D,E; Figure S3). Mantel tests for IBD detected a weak signal of IBD in the dataset with both species (Dataset 3.1; Mantel *r* = 0.12, *p* = 0.0098); however, IBD plots showed discrete patches of genetic differentiation irrespective of geographic differentiation corresponding to pairwise comparisons of *E. megasepala* and *E. tetrodonta* (Figure 5C). The detection of a comparatively much stronger signal of IBD in *E. tetrodonta* (Dataset 3.2; Mantel *r* = 0.32, *p* = 0.0001) suggests that genetic variation is strongly linked to geographic proximity in that species (Figure 5F). Given the discrete patches recovered in the IBD plot of Dataset 3.1 (Figure 5C), it is likely that the weak signal of IBD picked up for that dataset was simply an artefact of the very strong signal in *E. tetrodonta*. The results of *FEEMS* (Figure S4) were consistent across both of the tested grid cell sizes and found gene flow to be continuously reduced from north‐central Queensland across the Carpentarian Gap and into the Northern Territory, with isolated patches of low gene flow also occurring in some populations of *E. tetrodonta* in the Kimberley region, Western Australia. Aside from these isolated patches in Western Australia, a generally continuous region of higher gene flow was suggested to occur from the Kimberley, Western Australia through Arnhem Land, Northern Territory, and reaching the tip of Cape York Peninsula, Queensland. Hybrid indices and interclass heterozygosity statistics estimated in *triangulaR* did not provide clear evidence for hybridisation (Figure S5).

### 3.3 Outlier Locus Detection and Characterisation with GO Terms

**FIGURE 6:**
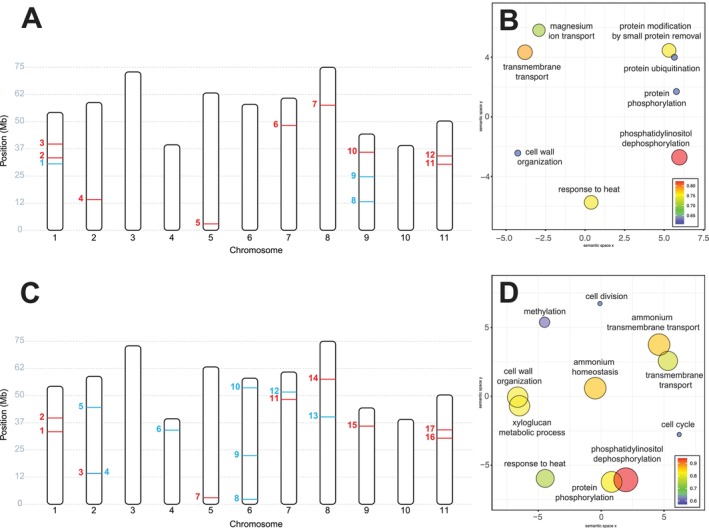
Outlier SNP locations and REViGO gene ontology visualisations for Dataset 4.1 (*Eucalyptus megasepala* and all *E. tetrodonta*; A, B) and Dataset 4.2 (*E*. *megasepala* and geographically proximal *E. tetrodonta* only; C, D). (A, C) Positions of outlier SNPs identified by *pcadapt* on the *E. grandis* genome (Myburg et al. 2014). SNPs in blue were identified uniquely for their respective dataset, while those in red were identified for both datasets 4.1 and 4.2. Numbers correspond to SNP information presented in Tables 1 and 2. (B, D) REViGO scatterplots showing Gene Ontology (GO) biological processes associated with outlier SNPs in semantic space (where axes have no intrinsic meaning and simply aid visualisation). For each plot, circles denoting GO terms are scaled and coloured according by the amount of allele fixation (i.e., the absolute difference in allele frequencies: |(AF_*E.meg*_—AF_*E.tet*_)/100|) of outlier SNPs associated with each term between *E. megasepala* and *E. tetrodonta* from 0 to 1, with higher values corresponding to higher fixation between species.

From the 994 SNPs present in Dataset 4.1, *pcadapt* identified 12 outliers among all samples of *E. megasepala* and *E. tetrodonta* (Table 1). From the 769 SNPs present in Dataset 4.2, which included only geographically overlapping samples of *E*. *megasepala* and *E. tetrodonta*, *pcadapt* identified 17 outliers (Table 2). For both datasets, *OutFLANK* and *BayeScan* did not detect any outliers. Of the 12 and 17 SNP outliers identified by *pcadapt* for each dataset, three SNP outliers were unique to Dataset 4.1, and eight SNP outliers were unique to Dataset 4.2 (Figure 6A,C). Outlier SNPs were distributed fairly evenly across the genome, being located on all but two chromosomes (3 and 10) of the *E. grandis* genome (Figure 6A,C).

**TABLE 1:** Details of SNPs identified by outlier analyses of Dataset 4.1. Numbers in the FigureIndex column relate to those in Figure 6A.

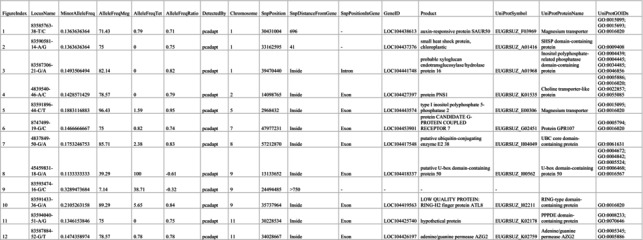

**TABLE 2:** Details of SNPs identified by outlier analyses of Dataset 4.2. Numbers in the FigureIndex column relate to those in Figure 6C.

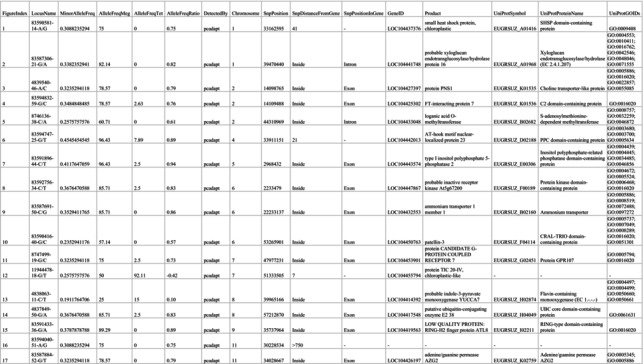

Outlier genes from Dataset 4.1 were involved in various biological processes related to cell wall organisation, response to heat, transmembrane transport, protein modification, and phosphatidylinositol dephosphorylation (Figure 6B). In particular, allele frequencies for genes linked to heat response, transport, and protein modification by small protein removal (such as ubiquitin or a ubiquitin‐like protein) were higher in *E. megasepala*; conversely, allele frequencies for genes linked to protein ubiquitination and phosphorylation were higher in *E. tetrodonta* (Figure 6B). Outlier genes from Dataset 4.2 were, for the most part, related to similar biological processes as those identified in Dataset 4.1, but were also involved in ammonium homeostasis (Figure 6D). Allele frequencies for genes linked to cell wall organisation (and xyloglucan metabolic process) were also higher for *E. megasepala*; this was opposite to allele frequencies being higher in *E. tetrodonta* for the same process in Dataset 4.1. Notably, the gene encoding FT‐interacting protein 7 was identified as an outlier in Dataset 4.2; however, the specific function of this gene in relation to flowering was lost by the broadness of the GO term associated with the *E. grandis* gene in UniProt (= GO:0016020: “membrane”). This limitation, which stems from uncertainty surrounding gene function and annotation in eucalypts, presumably also affected the level of GO information retrieved for other outliers, leading to the return of broad GO terms that lacked sufficient specificity to be of major use in assigning fine‐scale functions to outlier genes (Huntley et al. 2014; The Gene Ontology Consortium 2019).

### 3.4 Comparisons of Environmental Conditions and Flowering Times

**FIGURE 7:**
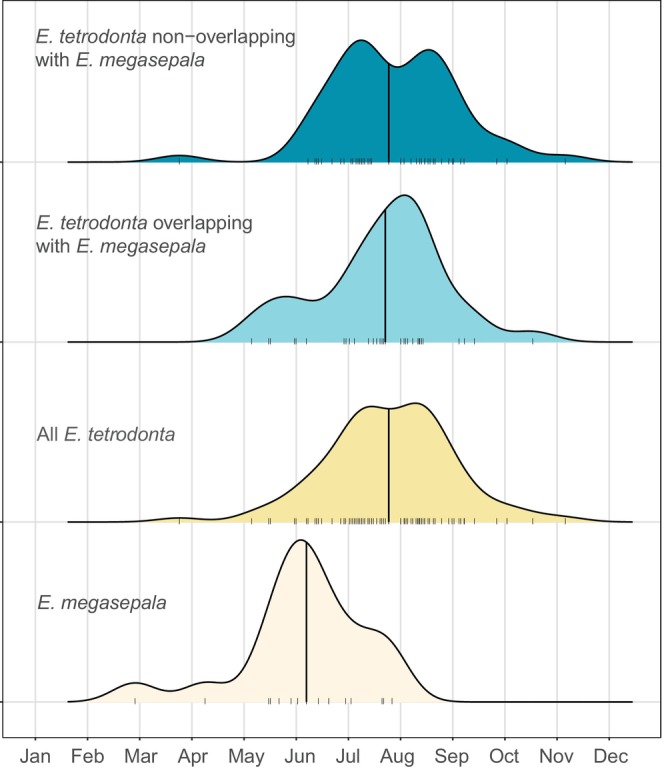
Flowering time differences between *Eucalyptus megasepala* and *E. tetrodonta* from cleaned AVH (2023) records represented by density curves. The bottom two curves include observations for all collections of *E. tetrodonta* and *E. megasepala*, while the top two curves include collections of *E. tetrodonta* from geographic ranges that are non‐overlapping and overlapping with *E. megasepala*, respectively. Vertical black lines show the median flowering time for each group. Ticks under density curves represent individual observations.

Flowering time was significantly different between collection records of the two species (Mann–Whitney U test; *p* = 3.75 × 10^−6^). The median flowering time of *E. megasepala* was 9 June, 48 days before the median for *E. tetrodonta* on 27 July (Figure 7). Flowering times for the geographically overlapping and non‐overlapping groups of *E. tetrodonta* were both significantly different from *E. megasepala* (overlapping Mann–Whitney U test, *p* = 2.75 × 10^−4^; non‐overlapping Mann–Whitney U test, *p* = 3.00 × 10^−6^), with the median flowering time for overlapping *E. tetrodonta* falling 2 days before that of the non‐overlapping group (25 July vs. 27 July). The difference between the flowering time of the two groups of *E. tetrodonta* was non‐significant (Mann–Whitney U test; *p* = 0.47).

The final set of bioclimatic variables included in the PCA were BIO3 (isothermality), BIO10 (mean temperature of the warmest quarter), BIO16 (precipitation of wettest quarter), and BIO17 (precipitation of driest quarter); these variables failed to discriminate any difference between *E. tetrodonta* and *E*. *megasepala* (Figure S6). As with the BIOCLIM variables, the soil variables were not able to distinguish the two species in northern Queensland.

## 4 Discussion

### 4.1 Strong Genetic Differentiation between *E. tetrodonta* and *E. megasepala* Provides Novel Insights Into the Evolutionary Dynamics of Eucalypt Speciation

Studies of eucalypt phylogenetics or population genetics routinely show signals of gene flow and/or introgression between closely related species, especially using plastid markers (Jackson et al. 1999; McKinnon et al. 1999, 2001, 2004, 2010; Pollock et al. 2013, 2015; Nevill et al. 2014; Fahey et al. 2021) but also using multilocus nuclear DNA datasets (Rutherford et al. 2018, 2019, 2021, 2023; Collins et al. 2019; Murray et al. 2019; von Takach Dukai et al. 2019; Fahey et al. 2022b). Our recent phylogenomic analyses of *Eucalyptus* subg. *Eudesmia*, to which our target species belong, also showed a legacy of gene flow throughout the evolution of the lineage (McLay et al. 2023). Such patterns are also commonly found in other large temperate tree genera, such as the oak genus, *Quercus*, which has been the subject of extensive study (McVay et al. 2017; Cavender‐Bares et al. 2015; Cannon and Petit 2020; Hipp et al. 2020). Given this background, the level of genetic differentiation observed between *E. tetrodonta* and *E. megasepala* is surprising. The fact that the two species are sisters to one another and overlap geographically in northern Queensland led us to hypothesise that we would detect some level of genetic admixture; in reality, there is almost none.

Although the ancestry plots from LEA (Figure 5A) and STRUCTURE (Figure S2) show some admixture between samples of *E. tetrodonta* and *E. megasepala*, this likely reflects the shared ancestry (sister relationship) of the two species, rather than recent genetic introgression between them (see Figure S5). For *E. tetrodonta*, the LEA plot shows mixed ancestry for a number of samples (up to ~13% “*E. megasepala*” ancestry), but there is no geographic pattern that could be considered consistent with recent introgression. For instance, one *E. tetrodonta* sample with mixed ancestry is from the zone of overlap between the two species (CANB 703268), but others are from outside the range of *E. megasepala* in Queensland (RMF 554.5, RMF 541.2, RMF 541.5, CANB 440141), the Northern Territory (CANB 535761) and Western Australia (MJB 2703) (see Figure 1 for locations of samples), making introgression an unlikely explanation for the admixture. The sample of *E. megasepala* (CANB 448844) showing more substantial admixture in LEA, but not in STRUCTURE, is explained as an artefact of the very high proportion of missing data (38% missing) for that sample. For *E. megasepala*, most samples show a proportion of mixed ancestry in both LEA and STRUCTURE plots, but the level of admixture is reasonably similar among all samples (0%–11% “*E. tetrodonta* ancestry” in LEA (excluding CANB 448844) and ~5%–27% in STRUCTURE). Given the close phylogenetic and phenotypic relatedness of *E. megasepala* and *E. tetrodonta*, in concert with high levels of heterozygosity and ILS in *Eucalyptus* species, this low but consistent pattern of mixed ancestry represents a background signal of the ancestral genetic pool of both species, rather than recent introgression.

The finding of strong genetic differentiation and limited evidence for an intergradation zone between sister species growing in adjacent habitats contrasts with the findings of many other studies on *Eucalyptus*. Extensive examples of intergradation zones where gene flow between closely related species occurs exist for *Eucalyptus* taxa growing both in adjacent geographic regions (e.g., *E. melanophloia* and *E. whitei* (Holman et al. 2011), *E. saligna* and *E. botryoides* (Passioura and Ash 1993) and Scribbly Gum spp. (Rutherford et al. 2023)), and abutting but contrasting habitat (e.g., *E. microcarpa* and *E. wimmerensis* (Fahey et al. 2022b), *E. rubida* and *E. aggregata* (Field et al. 2011), and the multiple examples of Robins et al. (2021). The differentiation between the two species examined here and the maintenance of strong species boundaries is likely the outcome of a complex interplay of environmental factors and biological speciation mechanisms (Levin 2006; Mallet et al. 2009; Givnish 2010), in addition to the lineage being far less speciose than the *Eucalyptus* lineages where large intergradation zones are typically observed (e.g., section *Eucalyptus*, section *Adnataria* and section *Glandulosae* which each hold > 50 species (Nicolle 2024)). Evidence for a lack of gene flow has also been observed between other sympatric and closely related species of *Eucalyptus*, including *E. salubris* and *E. ravida* (Binks et al. 2021), *E. dalrympleana* and *E. rubida* (Fahey et al. 2024), and *E. polybractea* and *E. viridis* (Fahey et al. 2022b). In combination with previously characterised ecological differences between the two species studied here, results from this research offer some insight into the factors potentially involved in the speciation and maintenance of clear genetic distinction between the two focal species and *Eucalyptus* species more broadly.

Species phenology is often an important ecological marker in distinguishing closely related species (e.g., in palms (Osborne et al. 2019), *Metrosideros* (Osborne et al. 2020), and irises (Osmolovsky et al. 2022)), and in limiting gene flow between sympatric or parapatric species or populations, including in eucalypts (Potts and Reid 1985; McGowen et al. 2001; Foster et al. 2007). Phenology was noted as a distinguishing character in the original description of *E. megasepala* (Bean 2006), and our analysis of herbarium specimen metadata revealed the extent of phenological differences between *E. tetrodonta* and *E. megasepala*; in particular, there is a strong separation of flowering times in Queensland, where the two species co‐occur (Figure 7). The presence of overlaps in the data may be due to seasonality shifts in the wet‐dry seasons in northern Australia that impact the onset of flowering in both species in different years, rather than an indication of actual flowering overlap. Notably, our investigation of outlier SNPs between the two species identified an SNP exhibiting perfect segregation between the two species, and that SNP resides within the gene FT‐interacting protein 7 (FTIP7), known to modulate flowering time in plants (Pin and Nilsson 2012; Yue 2012; Song et al. 2018). FTIP7 is a homologue of FTIP1, which mediates the movement of Flowering Locus T (FT) through the phloem. FT plays a central role in determining the onset of flowering (Pin and Nilsson 2012). Although we have not fully characterised differences in FTIP7 between the two species, our finding that one of the few segregating SNPs between the two species is associated with a gene that relates to a difference in phenology suggests this locus could be important in maintaining species boundaries.

Further ecological differentiation between the two species is found in subtle edaphic variation, even over extremely short distances where the two species can occur in the same vicinity on similar yet distinct substrate profiles. In Queensland, where the two species co‐occur, *E. tetrodonta* tends to occur on deeper, well‐drained, sandy, colluvial, and laterite soils and on downslope sites relative to *E. megasepala* (Franklin 2022). In contrast, *E. megasepala* occurs on harsher, upslope sites (on ridges, plateaus or outcrops) of lower quality, larger particle soils (Bean 2006; Franklin 2022). Our analyses based on environmental and soil data were not able to identify any differentiation, but this is likely due to the lack of fine‐scale granularity of the publicly available datasets used in the analyses. Geological mapping has identified Cape York Peninsula as a complex mosaic of ancient and more contemporary soils that are highly leached with low levels of nutrients (Wannan 2014). There is some evidence of heavy metals in the soils, such as manganese, gold, copper, and mineral ores such as bauxite, which may impact plant distributions in the area, but no current mapping of profiles is fine‐scale enough to differentiate between the soil preferences of the two species across their overlapping ranges (Biggs and Philip 1994; Denaro and Ewers 1995; Pain et al. 1995). Our outlier analyses also point to some loci associated with soil chemistry and heavy metal tolerances (e.g., ammonium and magnesium transport, Table 2). Slight differences in soil tolerances have been shown to impact distributions of other eucalypts over short ranges (e.g., Parsons 1969). If such a difference in soil chemistry is inhibiting either species, then it is possible that any potential hybrids are not well‐suited to survive on either of the substrates, leading to reinforcement of the differentiation between the two species (Rieseberg and Carney 1998; Givnish 2010).

Interactions between edaphic variables and climate are likely to have some bearing on variation in reproductive/flowering phenology in this system. The effects of temperature and rainfall have been shown to strongly influence the timing of flowering in eucalypts (Rawal et al. 2015a, 2015b), and many eucalypt species exhibit a flush of flowering following seasonal rainfall (Law et al. 2000). Northern Australia is defined by its strongly monsoonal climate, with a distinct, almost rainless dry season spanning 7–8 months followed by a torrential wet season (Woinarski et al. 2007), and relative to southern eucalypts, the reproductive biology of savannah species in northern Australia is likely to be even more tightly linked to this seasonal variation. Flowering of *E. tetrodonta* is observed to be seasonally linked, with bud formation and flowering occurring following the onset of the dry season (Setterfield and Williams 1996; Franklin 2022). Setterfield and Williams (1996) noted that peak seedfall of a Kakadu (Northern Territory) population of *E. tetrodonta* between 1992 and 1994 coincided with the first rains of the wet season and hypothesised that seeds that fall following the onset of rains experience increased germination success and reduced loss due to ant harvesting. In *E. tetrodonta* and *E. megasepala*, differences in flowering time may be related to differences in soil moisture and water retention as a consequence of soil or substrate variation, namely, the occurrence of *E. tetrodonta* on more fertile, deep soils of downslope sites and *E. megasepala* on shallower and less fertile, rocky upslope sites. Despite experiencing equivalent day lengths, temperatures and rainfall across their overlapping geographic ranges, the moisture retaining capacity of the soil/substrate is likely to vary considerably between the two species (i.e., the rocky upslope sites of *E. megasepala* could have lower soil water availability compared to *E. tetrodonta*). Some anecdotal evidence of this has been observed in the field; in November 2015 (i.e., late in the dry season), foliage on *E. megasepala* near Laura, Queensland was severely desiccated, whereas that of *E. tetrodonta* immediately downslope was not (DCF, personal observation). It is conceivable that the soil moisture conditions of *E. megasepala* may trigger an earlier flowering time relative to *E. tetrodonta*.

While we have some insight into the underlying mechanism(s) that likely maintain species boundaries and may have led to speciation, what is less clear is whether speciation of *E. tetrodonta* and *E. megasepala* occurred in close proximity as the two species now occur, or whether geographic separation between two populations allowed for differentiation prior to secondary contact. Sympatric or parapatric speciation is conceivable via local adaptation, potentially driven by subtle genetic changes at fine scales, for instance, through selection of traits associated with edaphic factors and reinforced by shifts in flowering time. Alternatively, vicariance and secondary contact and thus allopatric differentiation is also plausible, with barriers to gene flow, such as discrete flowering times and different ecological tolerances, developing in allopatry and preventing admixture on secondary contact. Given debate about the relative importance of sympatric versus allopatric speciation in plants (Fitzpatrick et al. 2008; Mallet et al. 2009; Bock et al. 2023), further studies of *E. tetrodonta* and *E. megasepala* could be worthwhile. These could better quantify ecological differentiation of the two species (given that environmental factors used here were largely overlapping), model their distributions under past climates and also study the extent of genetic differentiation, including in outlier loci, at a finer scale than done here. Inclusion of plastid DNA markers, which show widespread introgression in other eucalypt groups (McKinnon et al. 2010; Pollock et al. 2013, 2015; Nevill et al. 2014; Fahey et al. 2021) could also provide further insight into the extent of genetic differentiation between the two species.

The level of genetic differentiation that we detected at thousands of short SNP loci potentially points towards larger, more significant physical and genetic variation in the genomes of these two species that could be better detected with whole genome sequencing or longer DNA sequence reads. Genomic rearrangements and microsyntenic differences between the genomes of *Eucalyptus* species have recently been revealed to be a driver of genetic variation within the genus (Ferguson et al. 2023). Several papers by Ferguson et al. (2023, 2024b, 2024a) have shown how dynamic eucalypt genomes can be, with a high level of microsyntenic variation between species. Their studies focus on more distantly related species than *E. tetrodonta* and *E. megasepala*, so it is unclear how generalisable their findings may be to this speciation scenario. Given the outlier SNP findings might point towards important speciation mechanisms in plants (flowering time, soil substrates), there is also the possibility that differentiation between the two species relies on a handful of ‘barrier loci’ rather than genomic‐scale changes (Barton and Bengtsson 1986; Ravinet et al. 2017; Kautt et al. 2020). Further sampling of both specimens (populations more broadly, distinctly looking for possible hybrids, etc.) and genomic (whole genome and whole genome resequencing), together with better outgroup sampling, might lend itself to coalescent analyses and investigations of speciation with/without gene flow. This type of genetic sampling could be complemented with soil chemistry and moisture data for the edaphic profiles of both species, cytological data, common garden experiments and assessment of crossability to provide a more complete picture of differentiation and speciation between the two species.

### 4.2 Minimal Genetic Variation and Geographic Structure Exists in *E. tetrodonta* across Northern Australia

In contrast with the significant genetic differentiation observed between the two species in Cape York Peninsula, *E. tetrodonta* exhibits notably low genetic differentiation across its range throughout northern Australia (encompassing some 800,000 km^2^). Genetic variation in the species follows a clear IBD pattern (Figure 5), with most genetic structure occurring between samples collected from either side of the Carpentarian Gap. However, the specimens we sampled immediately adjacent to the Gap were genetic intermediates of the two genetic clusters, indicating possible admixture in the past between the eastern and western ranges of the species or the interruption of an IBD continuum caused by population extinction and a lack of suitable habitats (Figure 5).

The plains across northern Australia that *E. tetrodonta* occupies are old and geologically stable, but the climate and sea level have fluctuated significantly between periods of glacial maxima and warmer interglacial periods (Chivas et al. 2001; De Deckker 2001). During glacial maxima, sea levels dropped by up to 120–140 m, the Gulf of Carpentaria was reduced to a shallow lake, and land connections formed between Cape York and New Guinea across the Torres Strait (via the Torres Sill), and Arnhem Land and New Guinea across the Arafura Sea (via the Arafura Sill) (De Deckker 2001; Hope et al. 2004), providing vast tracts of newly revealed land and the opportunity for range expansions for a variety of organisms (Reeves et al. 2008; Edwards et al. 2017). During warm interglacial periods, sea levels were 5–8 m higher than today (Hope et al. 2004), resulting in a marine environment in the Gulf with the sea extending further inland than at present (Reeves et al. 2008). Together with glacial cycles and sea‐level changes, the region has also experienced short‐term patterns of aridity cycling (~2.6Ka), presenting another environment for expansion and adaptation (De Deckker 2001).

These historical environmental dynamics have undoubtedly impacted the biota of the region, and the Carpentarian Gap has been shown to be a barrier to many species distributions and the cause of major vicariance events in many taxa (Bowman et al. 2010; Edwards et al. 2017). While there are numerous biogeographic barriers that have been inferred across northern Australia (Edwards et al. 2017), the Carpentarian Gap is the only one that appears to have been somewhat of a barrier to gene flow in *E. tetrodonta*. Even so, our results suggest that it has not had a strong enough influence on the genetic structure in the species to produce discrete genetic groups from either side of the gap.

Bowman et al. (2010) suggested that the widespread distribution of *E*. *tetrodonta* could be explained by an ability to grow on well‐drained clay soils, such as on the plains of the Gulf of Carpentaria, as well as oligotrophic soils derived from sandstone and laterite that are present either side of the Carpentarian Gap on Cape York Peninsula and the Top End/Kimberley (Bowman et al. 2010). Certainly, the wide ecological tolerances of *E. tetrodonta* do not preclude it from possible range expansions onto the Torres and Arafura Sills during glacial periods; support for higher gene flow across the northern populations in our *FEEMS* analysis may reflect this. Despite these tolerances, the present‐day range of *E. tetrodonta* has a small east–west disjunction (of ~300 km; Figure 1) near the southeast corner of the Gulf of Carpentaria. The fact that the species is persisting on the periphery of the disjunction suggests that it is either at the edge of its ecological tolerances and this zone is acting as a narrow geographic border restricting gene flow or that the species simply hasn't yet reoccupied the area and reconnected the two genetic populations.

An alternative explanation for the relatively low genetic differentiation of *E. tetrodonta* across its large northern range (including across the Carpentarian Gap) is the long‐distance pollen transfer ability of a number of likely pollinator species. The little red flying fox ( *Pteropus scapulatus* ) has a widespread distribution across northern and eastern Australia, and eDNA studies have identified *E. tetrodonta* and *E. megasepala* flower nectar and pollen to be major components of their diet (Bradford et al. 2022). Observational and tracking data indicate that the little red flying fox is extremely mobile and is capable of long‐range movements of > 400 km per night or 3000–6000 km per year (Vardon and Tidemann 1999; Welbergen et al. 2020; Westcott et al. 2020) and is thus more than capable of the transfer of pollen across the Carpentarian Gap. Likewise, the nectarivorous varied lorikeet ( *Psitteuteles versicolor* ) is a documented visitor of *E. tetrodonta* flowers, with a wide distribution across northern Australia (Franklin and Noske 2000; ALA 2024). Flight distances are not known for the varied lorikeet; however, other lorikeet species are known to take daily flights to feeding sites up to 35 km away (Southerton et al. 2004), and flock influxes associated with eucalypt flowering events indicate flight distances of the varied lorikeet may be substantial (Woinarski and Tidemann 1991; D. Franklin pers. obs).

Within the eucalypts, patterns of distribution suggest that the Carpentarian Gap has played an important role in shaping extant diversity. Biogeographic analyses across the whole of *Eucalyptus* indicate two major phytogeographic regions across northern Australia, divided at the Carpentarian Gap; (1) monsoonal areas in Western Australia and Northern Territory and (2) more tropical areas of northern Queensland (González‐Orozco et al. 2014). Several other widespread species or sister‐species pairs in *E*. subg. *Eudesmia* (based on relationships inferred by McLay et al. 2023) are separated across the Carpentarian Gap including: *E. miniata* (west) and its sister *E. chartaboma* (east, on Cape York Peninsula); *E. lirata* (west) and its sister *E. similis* (east); and *E. phoenicea* , which has disjunct populations either side of the Carpentarian Gap, with those in the east considered by Nicolle (2024) to represent a distinct, undescribed species, referred to by the informal name “*Eucalyptus* sp. Battlecamp (Nicolle 1789)”. For all of these examples of eucalypt species that are closely related to *E. tetrodonta*, the Carpentarian Gap has seemingly played a key role in creating vicariant populations that have led to subsequent differentiation and speciation. The lack of strong phylogeographic structure in *E. tetrodonta* across the Gap could indicate either that *E. tetrodonta* is in the early stages of this process, or that the Gap does not represent a substantial barrier to the species because of its ecological tolerances or pollinator dispersal abilities.

Comparing this finding to other plant groups is difficult because biogeographic patterns across northern Australia using genetic methods are understudied in plants. Presently, our biogeographic understanding of the region is more heavily informed by studies on animals (including reptiles (Moritz et al. 2016; Potter et al. 2016; Fenker et al. 2024), mammals (Cremona et al. 2021; Potter et al. 2012, 2024; Umbrello et al. 2024), frogs (Catullo et al. 2014), and flies (Manawaduge et al. 2023)), which have generally found more discrete genetic groupings in northern Australia, associated with various biogeographic barriers, than we observed in *E. tetrodonta*. Further phylogeographic investigations of plant species with both wide and narrow distributions and diverse life history and reproductive traits are necessary in order to build a better understanding of plant biogeography in the northern Australian monsoon tropics using comparative phylogeography.

## Author Contributions

**Harvey K. Orel:** data curation (equal), formal analysis (lead), methodology (supporting), software (lead), visualization (lead), writing – original draft (lead), writing – review and editing (lead). **Todd G. B. McLay:** formal analysis (supporting), investigation (supporting), methodology (supporting), project administration (equal), resources (supporting), writing – original draft (lead), writing – review and editing (lead). **Daniel J. Murphy:** funding acquisition (supporting), resources (supporting), writing – review and editing (supporting). **David J. Cantrill:** conceptualization (equal), funding acquisition (equal), resources (supporting), writing – review and editing (supporting). **Frank Udovicic:** resources (supporting), writing – review and editing (supporting). **Patrick S. Fahey:** formal analysis (supporting), writing – review and editing (supporting). **Donald C. Franklin:** resources (supporting), writing – review and editing (supporting). **Donna Lewis:** resources (supporting), writing – review and editing (supporting). **Philip G. Docherty:** resources (supporting), writing – review and editing (supporting). **Adam White:** resources (supporting), writing – review and editing (supporting). **Michael J. Bayly:** conceptualization (equal), funding acquisition (equal), project administration (equal), resources (supporting), writing – original draft (supporting), writing – review and editing (supporting). **Rachael M. Fowler:** data curation (equal), formal analysis (supporting), investigation (lead), methodology (lead), project administration (equal), resources (lead), visualization (supporting), writing – review and editing (supporting).

## Conflicts of Interest

The authors declare no conflicts of interest.

## Supporting information


Figure S1.

Table S1.

## Data Availability

Supporting data and files for this manuscript are now publicly availablehere: https://doi.org/10.26188/27997571.v1.
