# Correction to “Genetic Differentiation of Geographically Overlapping Sister Species of *Eucalyptus* in Northern Australia”

**DOI:** 10.1002/ece3.71740

**Published:** 2025-07-04

Orel, H. K., T. G. B. McLay, D. J. Murphy, et al. 2025. “Genetic Differentiation of Geographically Overlapping Sister Species of *Eucalyptus* in Northern Australia.” *Ecology and Evolution* 15, no. 6: e71454. https://doi.org/10.1002/ece3.71454.

In the Acknowledgements section, the third sentence reads “We would like to thank the Australian Tropical Herbarium, Queensland Herbarium, Australian National Herbarium, University of Melbourne Herbarium, National Herbarium of Victoria, and Western Australian Herbarium for provision of leaf material for this study.” In this sentence, we accidentally omitted the acknowledgement of an additional non‐author contributor, the Northern Territory Herbarium. Instead, we would like this sentence to read “We would like to thank the Australian Tropical Herbarium, Queensland Herbarium, Northern Territory Herbarium, Australian National Herbarium, University of Melbourne Herbarium, National Herbarium of Victoria, and Western Australian Herbarium for provision of leaf material for this study.”

We apologize for this error.

