# Supplementary material for: Genetic Differentiation of Geographically Overlapping Sister Species of Eucalyptus in Northern Australia
Source: Ecol Evol. 2025 Jun 23;15(6):e71454. doi: 10.1002/ece3.71454 (PMC12183611; doi:10.1002/ece3.71454)
Supplement: Supplementary file 1 — Figure S1. [file ECE3-15-e71454-s002.pdf]

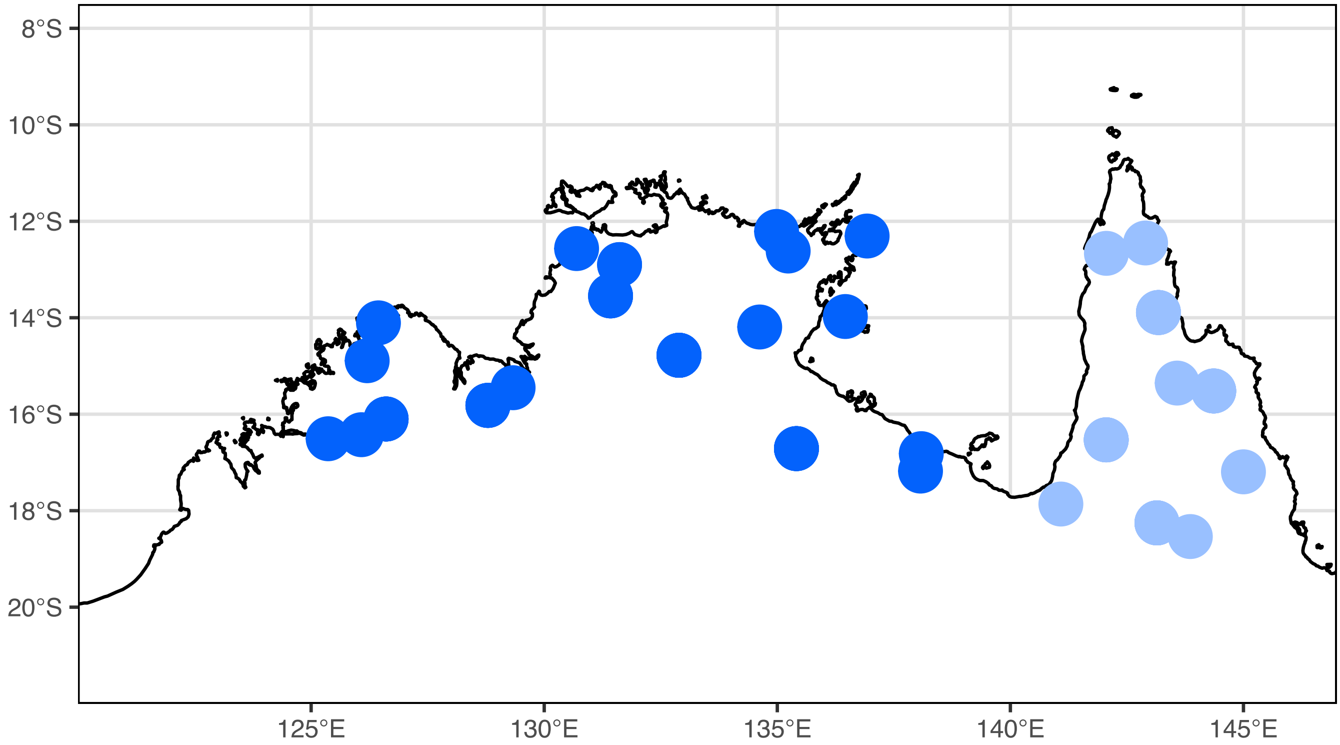

Figure S1. Map of *Eucalyptus tetrodonta* samples included in Dataset 3.2 coloured as per *k*-means clustering for $k = 2$.

A

K	Reps	Mean LnP(K)	Stdev LnP(K)	Ln'(K)	|Ln''(K)|	Delta K
1	20	-88463.045000	1.772443	—	—	—
2	20	-79290.085000	9.831275	9172.960000	8103.195000	824.226234
3	20	-78220.320000	1280.710721	1069.765000	1472.220000	1.149534
4	20	-78622.775000	275.241520	-402.455000	3878.030000	14.089553
5	20	-82903.260000	16589.631153	-4280.485000	8546.965000	0.515199
6	20	-78636.780000	3300.451774	4266.480000	12166.430000	3.686292
7	20	-86536.730000	40535.703217	-7899.950000	—	—

B

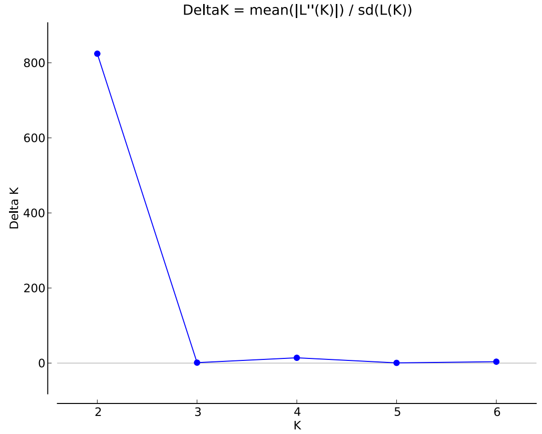

C

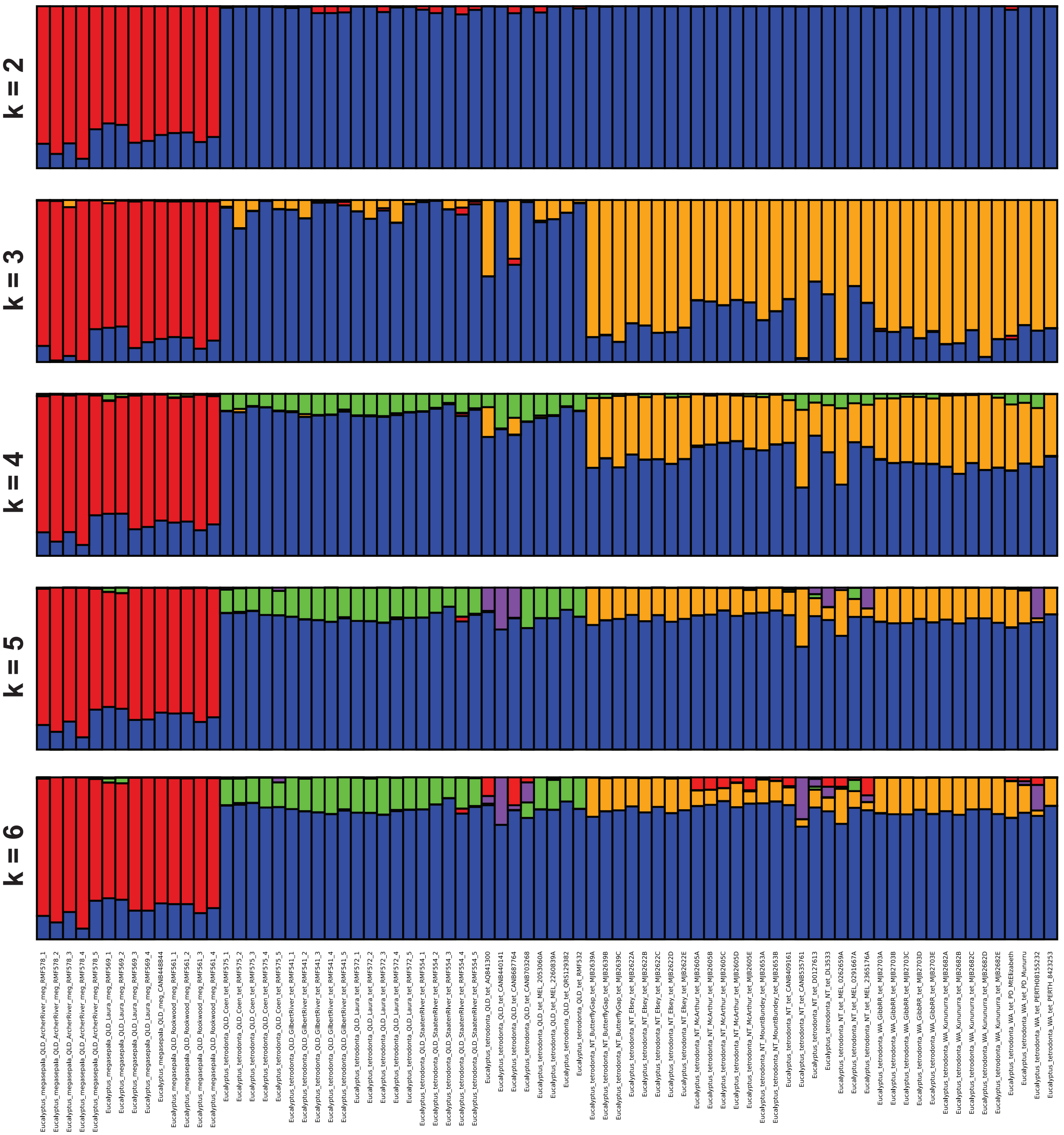

Figure S2. Structure analysis for Dataset 3.1. Population estimations were run for $k = 2–7$ (**A**) and optimal k identified as $k = 2$ (**B**). Ancestry proportions for $k = 2–7$ were then visualised as bar plots (**C**).

A

K	Reps	Mean LnP(K)	Stdev LnP(K)	Ln'(K)	|Ln''(K)|	Delta K
1	20	-62208.770000	1.690251	—	—	—
2	20	-60198.580000	17.819552	2010.190000	2961.780000	166.209569
3	20	-61150.170000	71.633424	-951.590000	1398.170000	19.518402
4	20	-60703.590000	169.641876	446.580000	3190.910000	18.809683
5	20	-63447.920000	12920.418521	-2744.330000	1612.250000	0.124783
6	20	-67804.500000	14418.840454	-4356.580000	5698.300000	0.395198
7	20	-66462.780000	13110.368529	1341.720000	—	—

B

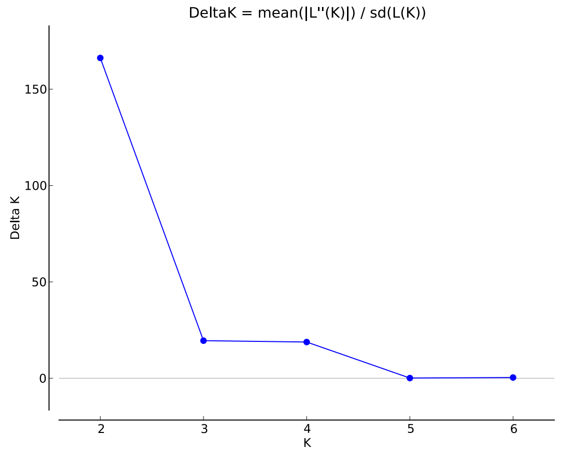

C

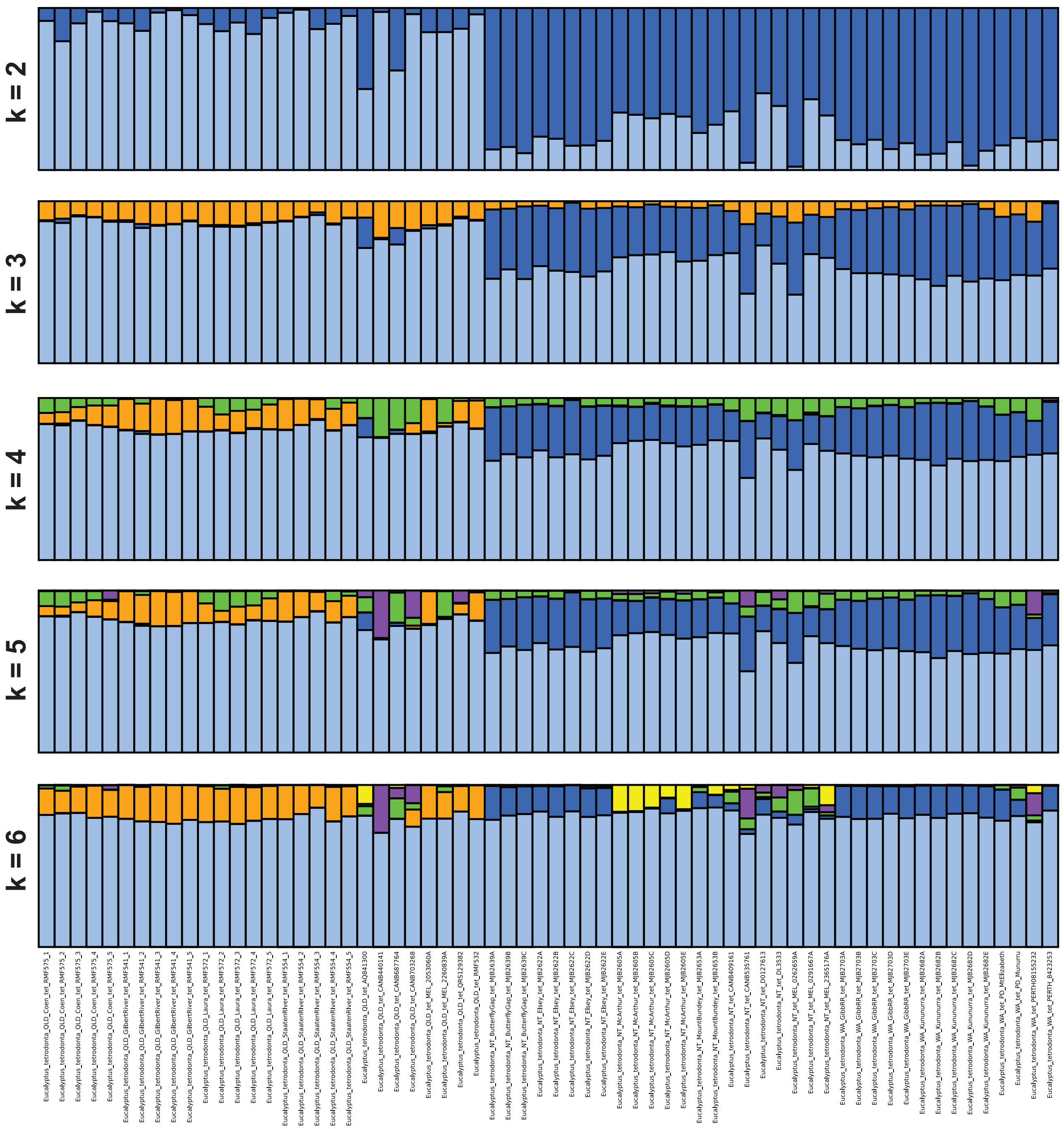

Figure S3. Structure analysis for Dataset 3.2. Population estimations were run for k = 2–7 (**A**) and optimal k identified as k = 2 (**B**). Ancestry proportions for k = 2–7 were then visualised as bar plots (**C**).

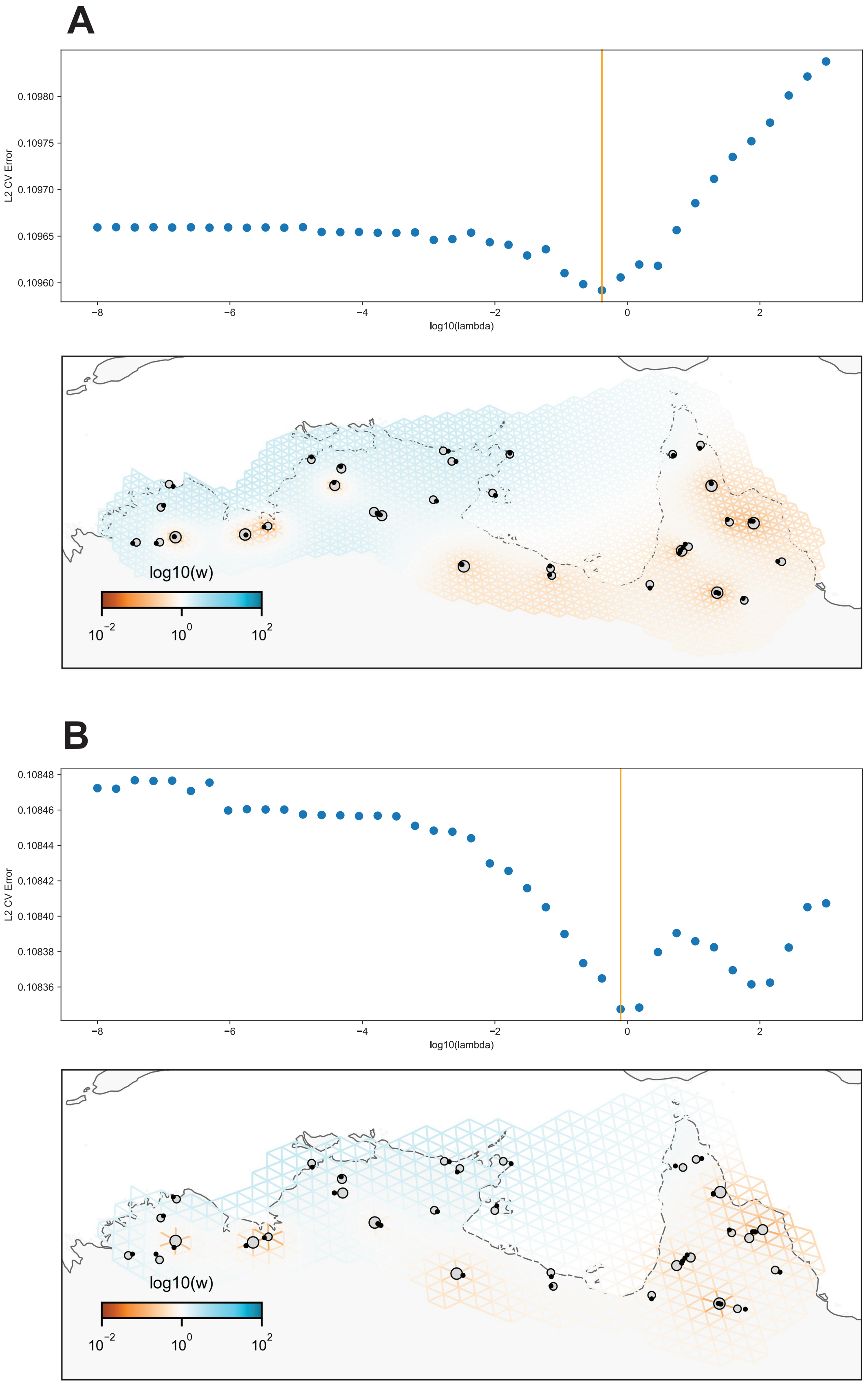

Figure S4. *FEEMS* analyses of Dataset 3.2 showing estimated effective migration surfaces at cell sizes of **(A)** 389 km^2 and **(B)** 1,557 km^2. Plots above maps show results of leave-one-out cross-validation for values of the smoothing regularisation parameter, λ, with the chosen values for λ being those with the lowest cross-validation error (λ = 0.41 for **A**, λ = 0.79 for **B**). Fitted parameters for these values of λ are shown on the map in log-scale, where lower effective migration is in orange and higher effective migration is in blue. Sample locations are denoted by small black points, and the nodes to which they are fitted are denoted by larger grey points; size of grey points is representative of the number of samples fitted to that node.

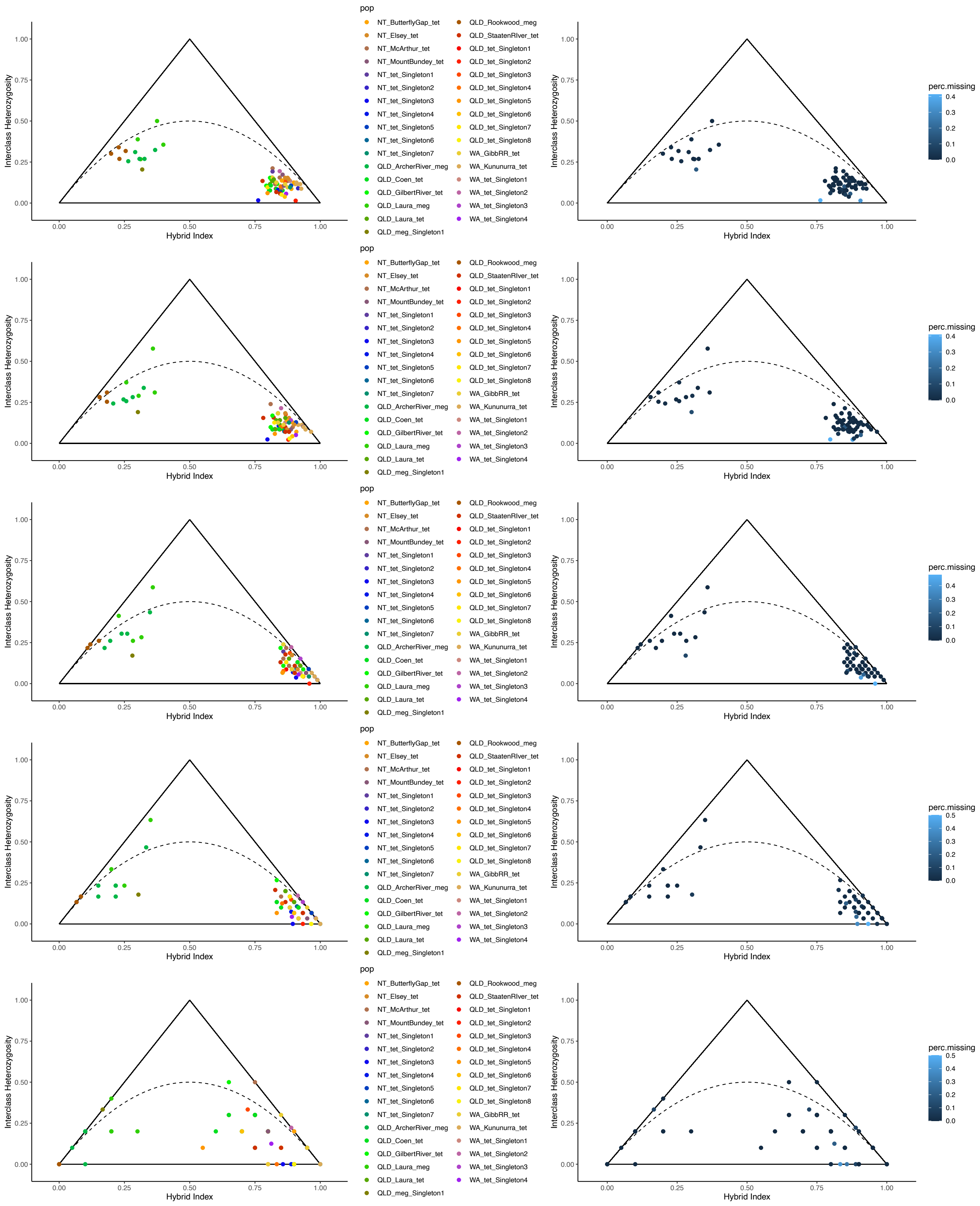

Figure S5. Hybrid indices calculated for allele frequency difference thresholds of 0.5, 0.6, 0.7, 0.8 and 0.9 on all populations from Dataset 4.1. Dashed line in triangle plot represents Hardy–Weinberg equilibrium.

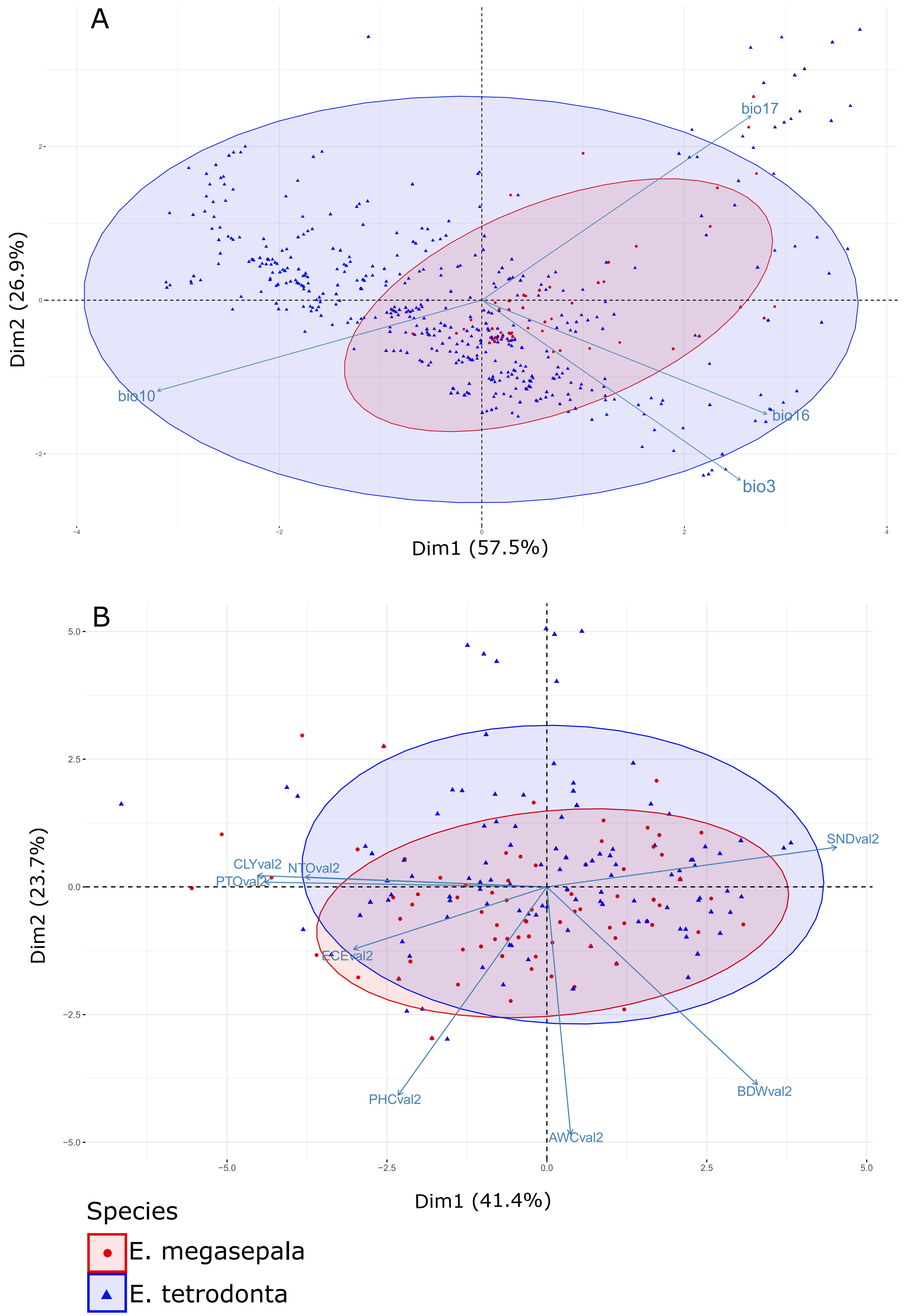

Figure S6. Environmental variables of *Eucalyptus megasepala* and *E. tetrodonta* from cleaned AVH (2023) data summarised using PCA. **(A)** BIOCLIM variables of all specimen data from throughout the range of *E. tetrodonta* (blue) and *E. megasepala* (red); the four BIOCLIM variables were BIO3 (isothermality), BIO10 (mean temperature of the warmest quarter), BIO16 (precipitation of wettest quarter), and BIO17 (precipitation of driest quarter). **(B)** Soil attribute variables for *E. megasepala* specimens and *E. tetrodonta* specimens from northern Queensland; the eight soil attributes included were AWC (available water capacity), BDW (bulk density), CLY (clay), ECE (effective cation exchange capacity), NTO (total nitrogen), pHc (pH CaCl2), PTO (total phosphorus), SND (sand). Ellipses are confidence intervals for each species calculated assuming a multivariate t-distribution.
