# Supplementary material for: Genetic Differentiation of Geographically Overlapping Sister Species of Eucalyptus in Northern Australia
Source: Ecol Evol. 2025 Jun 23;15(6):e71454. doi: 10.1002/ece3.71454 (PMC12183611; doi:10.1002/ece3.71454)
Supplement: Supplementary file 2 — Table S1. [file ECE3-15-e71454-s001.pdf]

Table S1. Details of samples used in this study. Herbarium abbreviations follow Index Herbariorum (https://sweetgum.nybg.org/science/ih/). Other abbreviations are as follows: DL, Donna Lewis; Hwy, highway; MJB, Michael J. Bayly; NT, Northern Territory; RMF, Rachael M. Fowler; WA, Western Australia; Qld, Queensland

**Collecting number, herbarium voucher**	**Locality**	**Latitude**	**Longitude**
Eucalyptus megasepala			
Liddle s.n., CANB 440890	Qld: Iron Mountain, S of Emuford	-17.5167	145.0167
Clarkson 9725, CANB 448844	Qld: 4 km from Dixie on road to Koolatah	-15.1167	143.2667
McDonald 7906, CANB 720345	Qld: c. 1 km N of Archer River crossing, Peninsula Development Road	-16.4267	142.9431
1346, MEL 2260840A	Qld: c. 6.5 km S of the Laura River (southern crossing), Peninsula Developmental Rd	-15.6269	144.4886111
RMF561.1, MELU D127964	Qld: Burke Development Rd, towards Chillagoe	-16.898503	144.2208653
RMF561.2, MELU D127964	Qld: Burke Development Rd, towards Chillagoe	-16.8941203	144.2175055
RMF561.3, MELU D127964	Qld: Burke Development Rd, towards Chillagoe	-16.8878504	144.2140491
RMF561.4, MELU D127964	Qld: Burke Development Rd, towards Chillagoe	-16.8843364	144.2121706
RMF569.1, MELU D127972	Qld: Peninsula Development Rd, S of Laura	-15.627168	144.4885977
RMF569.2, MELU D127972	Qld: Peninsula Development Rd, S of Laura	-15.623599	144.4777009
RMF569.3, MELU D127972	Qld: Peninsula Development Rd, S of Laura	-15.610806	144.4658586
RMF569.4, MELU D127972	Qld: Peninsula Development Rd, S of Laura	-15.6023124	144.4604466
RMF569.5, MELU D127972	Qld: Peninsula Development Rd, S of Laura	-15.5921899	144.453445

RMF578.1, MELU D127981	Qld: Peninsula Developmental Rd, just N of Mungkan Kandju NP	-13.561747	143.0191723
RMF578.2, MELU D127981	Qld: Peninsula Developmental Rd, just N of Mungkan Kandju NP	-13.5133041	142.986039
RMF578.3, MELU D127981	Qld: Peninsula Developmental Rd, just N of Mungkan Kandju NP	-13.5176887	142.9876332
RMF578.4, MELU D127981	Qld: Peninsula Developmental Rd, just N of Mungkan Kandju NP	-13.535414	143.0004894
RMF578.5, MELU D127981	Qld: Peninsula Developmental Rd, just N of Mungkan Kandju NP	-13.5565867	143.0155285
Eucalyptus tetrodonta			
NORM126, BRI AQ0841300	Qld: 185 km NW of Burketown on Wollogorang Station	-16.8175	138.0886111
8715, CANB 409161	NT: SE corner of Arafura Swamp	-12.6167	135.2333
Clarkson 8887, CANB 440141	Qld: 11.8 km E of Bromley on the track to Carron Valley	-12.45	142.9
Downey 313b, CANB 535761	NT: Cox Peninsula Rd between Bynoc Beach turn off and Mundorah	-12.5658	130.6944
Booth 4079, CANB 687764	Qld: Westmoreland Station, 28 km NW of Homestead	-17.1789	138.0722
Addicott 1795, CANB 703268	Qld: 12 km NE of Killnarney house	-15.3542	143.5786
Fell 9186, CANB 721291	Qld: Muralug (Prince of Wales Island)	-10.6567	142.1242
3905, DNA D0077682	NT: Bickerton Island; near Airport	-13.778566	136.1978308
Cowie 6968, DNA D0127613	NT: Little Bondi Beach; c 7km. SE of Yirrkala	-12.305789	136.9267157
21146, DNA D0187678	Qld: Westmoreland, near Little Amphitheatre	-17.3903	138.2697
DL 3533, DNA D0287005	NT: Groote Eylandt, near Central Hill	-13.97429	136.45937

Fox 182, MBA 3093.	Qld: W of Middle Park Homestead	-19.733	143.2
Cowie 7198, MEL 0262659	NT: Spirit Hills Con. Area, N of Nancys Gorge	-15.4506	129.3322
Clarkson 10563, MEL 0283971	Qld: 4.5 km SE of Mutchilba	-17.1333	145.25
10226, MEL 0291667	NT: Ramingining area, road to Dhabla	-12.2106	134.9842
Cowie 2956, MEL 1615886	NT: Cotton Island	-11.8	136.4833
3678, MEL 2020630	NT: Beside Allia Creek, c. 70 km S from Daly River Mission	-14.3097	130.6667
Adler s.n., MEL 2043372	NT: Nathan River Campsite	-15.3444	135.5478
839, MEL 2053060	Qld: Glenore, S of Normanton	-17.8622	141.0833
1355, MEL 2260839	Qld: Turn off to Stannery Hills, on Campbell road, c. 8.8 km S of Mutchilba	-17.1944	145
1430, MEL 2260841	Qld: c. 28.1 km E of Weipa towards Coen	-12.6644	142.0597
Cowie 13525, MEL 2365176	NT: Wongalara Wildlife Sanctuary; c 20 km E of homestead	-14.191667	134.6222222
MJB2605A, MELU D136693	NT: Carpentaria Hwy, c. 24.5 km W of intersection with Garrinjinny access road	-16.712583	135.410472
MJB2605B, MELU D136693	NT: Carpentaria Hwy, c. 24.5 km W of intersection with Garrinjinny access road	-16.7123052	135.410222
MJB2605C, MELU D136693	NT: Carpentaria Hwy, c. 24.5 km W of intersection with Garrinjinny access road	-16.7127774	135.410861
MJB2605D, TMELU D136693	NT: Carpentaria Hwy, c. 24.5 km W of intersection with Garrinjinny access road	-16.7130274	135.411694
MJB2605E, MELU D136693	NT: Carpentaria Hwy, c. 24.5 km W of intersection with Garrinjinny access road	-16.7127219	135.411722
MJB2622A, MELU D136694	NT: Stuart Hwy, N of Mataranka	-14.7780552	132.891389

MJB2622B, MELU D136695	NT: Stuart Hwy, N of Mataranka	-14.7590831	132.852139
MJB2622C, MELU D136694	NT: Stuart Hwy, N of Mataranka	-14.7583331	132.851778
MJB2622D, MELU D136694	NT: Stuart Hwy, N of Mataranka	-14.7126108	132.793722
MJB2622E, MELU D136694	NT: Stuart Hwy, N of Mataranka	-14.7118886	132.793333
MJB2639A, MELU D136696	NT: N of Pine Ck on Stuart Hwy, intersection with S end of Dorat Rd	-13.5466385	131.423083
MJB2639B, MELU D13669	NT: N of Pine Ck on Stuart Hwy, intersection with S end of Dorat Rd	-13.5468607	131.422111
MJB2639C, MELU D13669	NT: N of Pine Ck on Stuart Hwy, intersection with S end of Dorat Rd	-13.5458885	131.422389
MJB2653A, MELU D136697	NT: Arnhem Hwy, NW of Mary River crossing	-12.898194	131.61625
MJB2653B, MELU D136697	NT: Arnhem Hwy, NW of Mary River crossing	-12.8974163	131.615138
MJB2682A, MELU D136698	WA Victoria Hwy, E of Kununurra, near intersection with Crossing Falls Rd	-15.8170555	128.791972
MJB2682B, MELU D136698	WA Victoria Hwy, E of Kununurra, near intersection with Crossing Falls Rd	-15.8170278	128.792305
MJB2682C, MELU D136698	WA Victoria Hwy, E of Kununurra, near intersection with Crossing Falls Rd	-15.8171111	128.792805
MJB2682D, MELU D136698	WA Victoria Hwy, E of Kununurra, near intersection with Crossing Falls Rd	-15.8172778	128.793055
MJB2682E, MELU D136698	WA Victoria Hwy, E of Kununurra, near intersection with Crossing Falls Rd	-15.817	128.793527
MJB2703A, MELU D136699	WA: Gibb River Rd, gully E of road crossing of Gibb Range	-16.0991107	126.608055
MJB2703B, MELU D136699	WA: Gibb River Rd, gully E of road crossing of Gibb Range	-16.0987218	126.608361
MJB2703C, MELU D136699	WA: Gibb River Rd, gully E of road crossing of Gibb Range	-16.0985274	126.608778

MJB2703D, MELU D136699	WA: Gibb River Rd, gully E of road crossing of Gibb Range	-16.0986107	126.609444
MJB2703E, MELU D136699	WA: Gibb River Rd, gully E of road crossing of Gibb Range	-16.098944	126.609278
PD MtElizabeth, MELU D136700	WA: Mt Elizabeth Station	-16.424722	126.078611
PD Munurru, MELU D136701	WA: Munurru King Edward River Crossing Campsite	-14.8908331	126.201944
McCann 28, PERTH 8155232	WA: Anjo Peninsula, ca 44 km NW of Kalumburu and ca 7 km E of Truscott Airport, Kalumburu Aboriginal Reserve	-14.105556	126.4441667
H 639, PERTH 8423253	WA: Campground, Charnley River	-16.50854	125.36492
Connors 1430, QRS 129382	Qld: C. 28.1 km E of Weipa towards Coen	-12.664	142.059722
RMF532, MELU D127935	Qld: Located along track to Wundu campground, Canyon Resource Reserve	-18.536083	143.8605343
RMF541.1, MELU D127944	Qld: Gulf Development Rd between Georgetown and Croydon, near Gilbert River	-18.252797	143.1443704
RMF541.2, MELU D127944	Qld: Gulf Development Rd between Georgetown and Croydon, near Gilbert River	-18.2512529	143.1354973
RMF541.3, MELU D127944	Qld: Gulf Development Rd between Georgetown and Croydon, near Gilbert River	-18.2442048	143.1246183
RMF541.4, MELU D127944	Qld: Gulf Development Rd between Georgetown and Croydon, near Gilbert River	-18.22632	143.0891696
RMF541.5, MELU D127944	Qld: Gulf Development Rd between Georgetown and Croydon, near Gilbert River	-18.2233281	143.058771
RMF554.1, MELU D127957	Qld: Burke Development Rd, bank of Staaten River between Normanton and Chillagoe	-16.531683	142.0556893
RMF554.2, MELU D127957	Qld: Burke Development Rd, bank of Staaten River between Normanton and Chillagoe	-16.4460216	142.0795043
RMF554.3, MELU D127957	Qld: Burke Development Rd, bank of Staaten River between Normanton and Chillagoe	-16.4407525	142.0816571

RMF554.4, MELU D127957	Qld: Burke Development Rd, bank of Staaten River between Normanton and Chillagoe	-16.3377805	142.160687
RMF554.5, MELU D127957	Qld: Burke Development Rd, bank of Staaten River between Normanton and Chillagoe	-16.2168628	142.2341216
RMF572.1, MELU D127975	Qld: Peninsula Developmental Rd, approx. 10 km N of Laura	-15.51833	144.3623002
RMF572.2, MELU D127975	Qld: Peninsula Developmental Rd, approx. 10 km N of Laura	-15.5143174	144.355706
RMF572.3, MELU D127975	Qld: Peninsula Developmental Rd, approx. 10 km N of Laura	-15.5121229	144.3498061
RMF572.4, MELU D127975	Qld: Peninsula Developmental Rd, approx. 10 km N of Laura	-15.5057972	144.2885813
RMF572.5, MELU D127975	Qld: Peninsula Developmental Rd, approx. 10 km N of Laura	-15.4986355	144.2783672
RMF575.1, MELU D127978	Qld: Peninsula Development Rd, few km N of Coen	-13.891601	143.1760939
RMF575.2, MELU D127978	Qld: Peninsula Development Rd, few km N of Coen	-13.8871646	143.1731649
RMF575.3, MELU D127978	Qld: Peninsula Development Rd, few km N of Coen	-13.8806482	143.1723821
RMF575.4, MELU D127978	Qld: Peninsula Development Rd, few km N of Coen	-13.873726	143.1724817
RMF575.5, MELU D127978	Qld: Peninsula Development Rd, few km N of Coen	-13.8681984	143.1695329
**Outgroups**			
Eucalyptus chartaboma			
RMF521, MELU D127924	Qld: Undara Resort	-18.203221	144.5748525
Eucalyptus eudesmioides			
BB524, MELU D127739	WA: ~42 km N Galena Bridge, 10 km E of West Coastal Hwy	-27.4912	114.85045

Eucalyptus similis			
RMF531, MELU D127934	Qld: Canyon Resource Reserve	-18.542834	143.8865595
